# Genome Study of a Novel Virulent Phage vB_SspS_KASIA and Mu-like Prophages of *Shewanella* sp. M16 Provides Insights into the Genetic Diversity of the *Shewanella* Virome

**DOI:** 10.3390/ijms222011070

**Published:** 2021-10-14

**Authors:** Katarzyna Bujak, Przemyslaw Decewicz, Joanna M. Rosinska, Monika Radlinska

**Affiliations:** Department of Environmental Microbiology and Biotechnology, Institute of Microbiology, Faculty of Biology, University of Warsaw, Miecznikowa 1, 02-096 Warsaw, Poland; k.bujak@biol.uw.edu.pl (K.B.); p.decewicz@uw.edu.pl (P.D.); jm.rosinska@student.uw.edu.pl (J.M.R.)

**Keywords:** bacteriophage, prophage, cold-active, extreme environment, *Shewanella*, comparative genomic, RNA polymerase, A1 protein, dam-like methyltransferase, introns, Mu-like phage

## Abstract

*Shewanella* is a ubiquitous bacterial genus of aquatic ecosystems, and its bacteriophages are also isolated from aquatic environments (oceans, lakes, ice, and wastewater). In this study, the isolation and characterization of a novel virulent *Shewanella* phage vB_SspS_KASIA and the identification of three prophages of its host, *Shewanella* sp. M16, including a mitomycin-inducible Mu-like siphovirus, vB_SspS_MuM16-1, became the starting point for comparative analyses of phages infecting *Shewanella* spp. and the determination of their position among the known bacterial viruses. A similarity networking analysis revealed the high diversity of *Shewanella* phages in general, with vB_SspS_KASIA clustering exclusively with *Colwellia* phage 9A, with which it forms a single viral cluster composed of two separate viral subclusters. Furthermore, vB_SspS_MuM16-1 presented itself as being significantly different from the phages deposited in public databases, expanding the diversity of the known Mu-like phages and giving potential molecular markers for the identification of Mu-like prophages in bacterial genomes. Moreover, the functional analysis performed for vB_SspS_KASIA suggested that, despite the KASIA host, the M16 strain grows better in a rich medium and at 30 °C the phage replication cycle seems to be optimal in restrictive culture conditions mimicking their natural environment, the Zloty Stok gold and arsenic mine.

## 1. Introduction

Extremophile and extremotolerant organisms are found in many environments, including toxic waste, acid mine drainage, hypersaline and alkaline lakes, high pressures, polar or hot ecosystems, and arsenic-rich environments. Like other organisms, extremophiles serve as hosts for viral replication, and thus the exploration of niches with seemingly harsh life conditions permits us to successfully track the presence of viruses [1,2,3]. Moreover, it has been established that bacterial viruses (bacteriophages, or phages for short) in extreme niches, as in any other ecosystem, shape the composition and evolution of bacterial communities [4].

The organization of phage genomes isolated from such environments is generally similar to the genomes of other viruses infecting bacteria, but their encoded proteins often have unique amino acid sequences conferring stability and endowing these viruses with the ability to maintain infectivity under extreme conditions [1,5].

Before now, a number of bacteria–phage systems from various extreme environments have been described, e.g., the cold-active phage 9A of *Colwellia psychrerythraea* 34H, isolated from a 128-m-deep Arctic nepheloid layer [6]; inducible myovirus AcaML1 from the extreme acidophile *Acidithiobacillus caldus* ATCC 51,756 [7]; and several others infecting halobacteria, e.g., *Deleya halophile*, *Halomonas halophile*, and *Salinivibrio costicolan* [8].

An example of an extreme environment is the closed Zloty Stok gold and arsenic mine, located in Western Sudetes, Poland. This mine comprises over 300 km of underground passages on 21 levels. One of the most well-preserved galleries is the Gertruda Adit (the sample collection site of this study). It has been observed that within this Adit there are constant conditions of a low air temperature (10–11 °C), a reduced concentration of oxygen (17.2%), high humidity (~100%), and slightly alkaline water (pH 7–8) [9,10]. The most distinct extreme feature of this environment is the high concentration of numerous heavy metals, e.g., the arsenic content is ~5000–6800 mg/L for mats and ~3000–7000 μg/L for the mine waters [11], while typical background concentrations of arsenic do not exceed 100 mg/kg in soil and 10 μg/L in freshwater [10]. This unique ecosystem is inhabited by various microorganisms that are capable of growing in its specific and nutrient-limited conditions, and are well adapted to elevated concentrations of As and other toxic compounds. They are organized in spatially structured complex systems—biofilms covering the corridor walls and ceiling, and mats at the bottom sediments of mine water [11,12,13].

So far, four active phages from this exceptional ecological niche have been described, but all of them are temperate ones. Besides three mitomycin-induced prophages, one was an environmental virus isolated from rock biofilm. These were ΦO23A of *Aeromonas* sp. O23A [14], MuSsp1_O23S of *Shewanella* sp. O23S [15], vB_SspS_OS31 of *Serratia* sp. OS31, and the *Serratia* phage vB_SspM_BZS1 [16]. In the present study, we report on the isolation and characterization of the first virulent phage originating from the microbial mats of the Zloty Stok gold and arsenic mine, i.e., the vB_SspS_KASIA phage, which was obtained using the indigenous bacteria *Shewanella* sp. M16 as the host.

Until now, the genomes of one filamentous phage SW1 [17] and 13 *Caudovirales* phages infecting *Shewanella* spp.—a ubiquitous bacterial genus of aquatic ecosystems and a key organism for various biotechnological applications, e.g., bioremediation [18]—have been deposited in the public sequence repositories; they are available in the NCBI GenBank database (Table S1). Among them, eight phages belong to the family *Myoviridae*, three to *Siphoviridae*, one to *Podoviridae*, and one to *Chaseviridae*. We also noticed that, despite the names indicating a different bacterial host, *Vibrio*, six viruses of the Nahant Collection [19] were isolated using *Shewanella* spp. as the host, which would increase the number of known *Shewanella* phages to 19, with five more *Siphoviridae* and one more *Myoviridae* (Table S1). Moreover, there are also four prophages of *Shewanella oneidensis* MR-1, i.e., MuSo1, MuSo2, LambdaSo, and CP4So (although their genomes have not been extracted and deposited separately), of which the impact on the biofilm formation and cold tolerance of their host was thoroughly studied [20,21].

All of the known *Shewanella* phages were isolated from various aquatic environments, i.e., fresh water, seawater, wastewater, and even from Arctic sea ice [22]. However, the KASIA phage is unique, as it is the first virulent *Shewanella* virus isolated from microbial mats, and also because it comes from an ecological niche where the parasite, together with its host, must thrive under multiple extremes. Moreover, to our best knowledge, KASIA is the first *Shewanella* virus originating from an extreme environment that is characterized not only in terms of morphology and infection kinetics but also genomics. In this study, we also demonstrate that this virus is able to propagate in a narrow range of external factors (e.g., temperature), as compared to the greater flexibility of its host, *Shewanella* sp. M16.

## 2. Results and Discussion

### 2.1. Identification and Characterization of Shewanella sp. M16

The *Shewanella* sp. M16 was isolated from the microbial mats of the Gertruda Adit in the Zloty Stok gold and arsenic mine (SW Poland) in September 2018 using a method described in Section 3.1. The genomic DNA of *Shewanella* sp. M16 was isolated and sequenced. The reconstruction of its genome resulted in 180 contigs with a total length of 5,284,315 bp and 45% average GC content. Because M16 originated from the same environment as the arsenic-metabolizing O23S strain described in 2015 [15,23], we compared the two genomes. As a result, we found that both are nearly identical, except for two additional contigs (NZ_JAGTUL010000052 and NZ_JAGTUL010000080) in the M16 strain, on which a set of genes coding the type IV secretion system and replication module were identified.

Analyses performed on LB and R2A showed that *Shewanella* sp. M16 can grow at various temperatures (4–30 °C), with the optimum being 20–30 °C in both media (Figure S1, and see 2.7.2). Moreover, *Shewanella* sp. M16 multiplied about three times faster in LB than in R2A liquid medium (Figure S1). Similar tests of *Shewanella* sp. O23S performed on a LB medium showed that it can also grow in a broad range of temperatures, with the optimum being 22 °C [23].

### 2.2. Identification and Characterization of the vB_SspS_KASIA Phage

**Figure 1:**
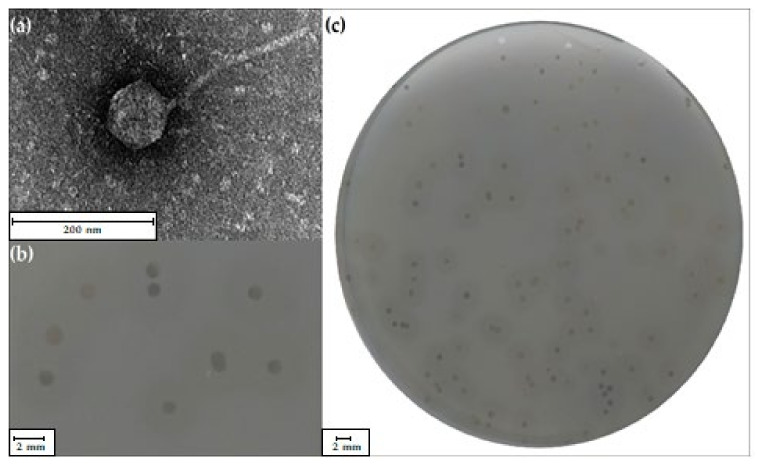
Characteristics of the virion (**a**) and plaques (**b**,**c**) of vB_SspS_KASIA. The scale bar in the transmission electron microscopy (TEM) image represents 200 nm. The scale bar in (**b**) and (**c**) images represents 2 mm.

The *Shewanella* sp. M16 strain was used to search for bacteriophages present in microbial mat samples of the Zloty Stok gold mine, which resulted in the isolation of the vB_SspS_KASIA virus, which was later called KASIA. Electron micrographs of negatively stained KASIA virions showed icosahedral heads of 91 ± 1 nm in diameter and a long, noncontractile tail 225 ± 2 nm in length and 12 ± 0.2 nm in width (Figure 1a). This morphology indicated that KASIA could be classified in the *Siphoviridae* family.

The measurement of 10 KASIA plaques formed on a *Shewanella* sp. M16 lawn grown on R2A agar plates at 10 °C revealed that their size was 2 ± 1 mm in diameter. These plaques were surrounded by an opaque-looking halo zone (5 ± 1 mm in diameter) that appeared 24 h after the plaque formation and increased upon prolonged incubation (Figure 1b,c). The halo zone hints at the presence of a virion-associated enzyme, depolymerase, which degrades the bacterial exopolysaccharide layer [24].

### 2.3. Genomic Analysis of the vB_SspS_KASIA Phage

The analysis of the phage KASIA’s genome sequence showed that it had a length of 91,102 bp, making it the largest known *Shewanella* phage genome of the *Siphoviridae* family. Four *Shewanella* phages (1/4, 1/40, Thanatos-1, Thanatos-2) have larger genomes, but they belong to the *Myoviridae* family. Phage genomes vary considerably in size, from ca. 5 to 500 kb. Based on an analysis of 500 phage genomes, Hatfull concluded that there was not a uniform distribution of genome sizes across that spectrum [25]. Interestingly, based on the analysis of a 30-fold-larger spectrum of sequence data (14,470 complete phage genome sequences, Figure S2), our conclusions were very similar. The majority of the known phages have genomes in the size range of 30–50 kbp; the second group have genomes smaller than 10 kbp, and the third group are those with 120−170 kbp genomes. Although we are aware that this overall distribution might not reflect the phage genome length distribution in nature, we found that a genome length of about 91 kbp (like KASIA) was quite rare in known bacterial viruses.

The KASIA genome GC content is 51.6%, which is 6% higher than that of the M16 strain. The KASIA genome was predicted to contain 145 putative genes. The annotation and organization of the KASIA genome are provided in Table S2. The function was assigned to 57 ORFs (39% of the total number of genes), while the remaining 88 ORFs were classified as hypothetical proteins. Among them, 54 shared no significant similarity with the proteins in GenBank. The analysis of the genome sequence coverage and the application of the PhageTerm tool [26] revealed the presence of 6912-bp-long non-permuted direct terminal repeats at the ends of the KASIA phage genome (File S1), yielding a 98,014 bp physical genome. Most phages with exact terminal repeats have short terminal repeats up to a few hundred base pairs long (e.g., T3 and T7 have 160 and 231 base repeats, respectively). Several phages have long exact terminal direct repeats. Among them, the best studied are *Escherichia coli* siphovirus T5 and *Bacillus subtilis* myovirus SPO1, of which the repeats are 10,139 and 13,185 bp in length, respectively [27,28]. The mechanism by which long repeats are generated is not known, but as with the short, exact repeat phages, the appearance of only one copy of the terminal repeat between genomes in replication-generated concatemers suggested that the repeat region is duplicated during packaging [29]. As long non-permuted direct terminal repeats are a feature of myo- and siphoviruses with large (over 120 kb) genomes [30], the KASIA phage, with its 91.1 kb genome, would be the smallest among them.

**Figure 2:**
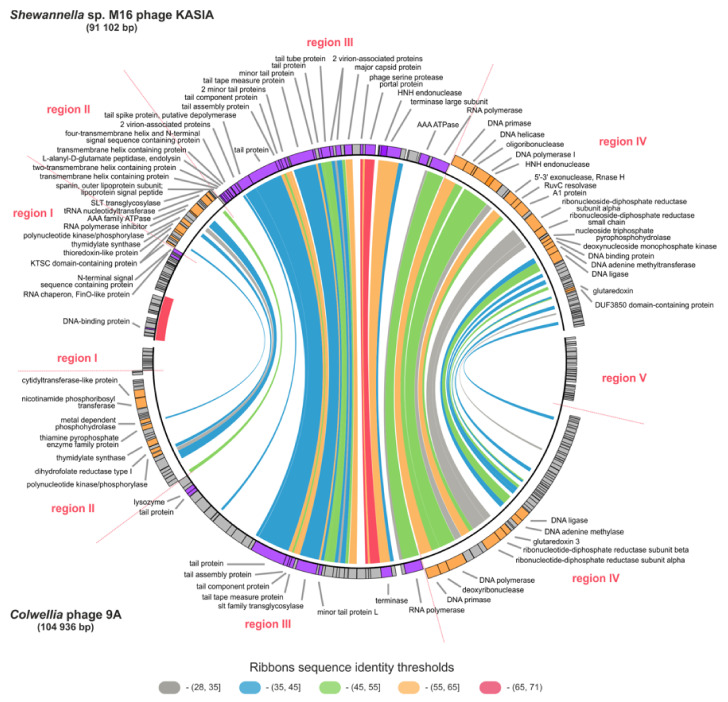
Comparison of the KASIA phage with *Colwellia* phage 9A. The similarities were based on all-against-all blastp searches, and are reflected by the ribbons connecting the ideograms. Their colors reflect the scale of the similarity between the encoded proteins, as indicated in the legend. The anchors of the ribbons correspond to the location of the protein-coding genes.

At the nucleotide sequence level, the KASIA phage shared no significant similarity with bacteriophages from the NCBI database. However, at the amino acid sequence level, it shared similarity with *Colwellia* phage 9A (Figure 2), which is also a virus infecting a gamma-proteobacteria isolated from cold, aquatic environments, and was described as the most extreme of the cold-active phages known [6]. The *Colwellia* phage 9A genome is longer (104,936 bp) than the KASIA genome, and it has a significantly lower GC content (37.0%) but the same %GC as its host, *Colwellia psychrerythraea* strain 34H [31]. Its annotation, dating back to 2013, revealed the presence of 31 (21%) gene products related to proteins of which the function was already known [32]. The majority of them had counterparts in the KASIA genome, and were referred to in the detailed comparisons described below. No gene encoding tRNA was detected in KASIA, as was the case in the *Colwellia* phage 9A genome.

In the KASIA genome, we distinguished four regions within which genes are transcribed in one direction. Region I contains early genes, region II comprises nucleotide metabolism genes, region III contains late genes (structural, capsid assembly, entry/lysis), and region IV is composed primarily of replication genes. The *Colwellia* phage 9A genome has a similar organization, although its assumed orientation split one block of early genes into two [32]. Moreover, 47 KASIA proteins are similar to their counterparts in *Colwellia* phage 9A (Table S2). Most of them are encoded in structural and replication modules (Figure 2).

In KASIA phage region I, we distinguished 34 genes that are likely expressed during an early stage of infection. Most of them encode hypothetical proteins with unassigned functions. We were only able to annotate the function of one protein. Within the KASIA_p031 sequence, a FinO_conjug_rep domain (cd00236) was identified. Interestingly, FinO-like proteins are primarily encoded in plasmids, but rarely in phage genomes, e.g., QQK87997.1 of *Salmonella* phage pSal-SNUABM-01 g (39.02% sequence identity). It was demonstrated that the FinO protein, a part of the two-component system FinOP, encoded by *Escherichia coli*, acts as an RNA chaperone [33]. The analysis of the *KASIA_p031* gene’s surroundings did not indicate that the putative products of the neighboring genes are similar to the FinP protein. Therefore, if the KASIA_p031 protein acts as an RNA chaperone, its mechanism of action is presumably different from that of the FinO protein of *E. coli*.

Within region II, seven genes encoding proteins potentially involved in nucleotide metabolism were identified. We found that the KASIA_041 protein contains a thioredoxin domain (cd02947). The genes encoding thioredoxins are found in phage genomes; however, the product of the *KASIA_041* gene did not share similarity with any phage thioredoxins, but showed similarity with bacterial ones, e.g., the thioredoxin of *Maribacter arcticus* (WP_079511329.1; 40.58% sequence identity). In the putative product of the *KASIA_042* gene, we observed a FAD-dependent thymidylate synthase domain (cd20175). Thymidylate synthases are found in almost all living species, including bacteriophages such as the *Escherichia* T4 phage [34]. In infected cells, thymidylate synthase encoded by the T4 phage resides in a structured complex of viral enzymes involved in deoxynucleoside triphosphate synthesis [35]. Thymidylate synthase converts deoxyuridylic acid (dUMP) into thymidylic acid (dTMP) [36]. The KASIA_042 protein revealed a similarity with its counterpart in the *Pseudomonas* phage PaoP5 (YP_009224816.1; 41.94% sequence identity) [37] and the thymidylate synthases of other *Pseudomonas* phages. Another protein, the putative KASIA_p044 polynucleotide kinase/phosphorylase, showed similarity with its counterpart, *Colwellia* phage 9A (YP_006489218.1; 38.26% sequence identity). An HHpred search of KASIA_p047 identified a hit for bacterial RNA polymerase inhibitor (97.13% probability). It was demonstrated that some phages, such as *Escherichia coli* phage T7, encode an inhibitor of the host’s DNA direct RNA polymerase (RNAP) that binds to its β′ subunit downstream jaw domain and prevents open promoter complex formation by Eσ70, which allows it to shut off the host’s RNAP and use viral RNAP to transcribe the middle and late genes of the phage [38]. Such a function of KASIA_p047 is quite likely, as the KASIA phage (similarly to *Escherichia coli* phage T7) encodes its own RNAP (see below). Based on the HHpred searches, the *KASIA_p052* gene seems to encode tRNA nucleotidyltransferase (99.86% probability). tRNA nucleotidyltransferases are involved in translation, and are responsible for generating and maintaining the nucleotide triplet CCA at the tRNA 3′ end. Such enzymes are encoded in the phage genomes of, e.g., *Pectobacterium* phages PP47, PP81, and Q19 [39]. We also found that KASIA_p035 and KASIA_p038 proteins share 42% sequence identity, and both contain KTSC domains. The function of this domain is unknown, but it is postulated that it could be an RNA-binding domain (PF13619) because it was previously discovered at the C-terminus of some lysyl-tRNA synthetases [40].

In region III, two functional modules can be distinguished: structural and lytic. The genes of the lytic module are located downstream from the genes encoding structural proteins. However, the first gene of the 5′ end of this region (*KASIA_p096*) encodes DNA direct RNA polymerase, which showed similarity to the RNAP of *Colwellia* phage 9A (51.77% sequence identity) and RNAPs of other phages, such as *Mesorhizobium* phage vB_MloP_Lo5R7ANS (NC_025431.1) or *Ralstonia* phage RsoP1EGY (NC_047946.1) (24–28% sequence identity). Generally, the transcription of phage genes is carried out by a complex, multicomponent bacterial RNAP; however, in some bacteriophages (e.g., *Escherichia* phage T7), most genes are transcribed by a single-subunit phage RNAP. RNAP is the hallmark of the *Autographiviridae* family [41]. In the *Autographiviridae* family, due to the localization of the gene encoding the RNAP, two subgroups can be distinguished: T7-like and ϕKMV-like. In T7-like phages, a gene encoding RNA polymerase is transcribed during an early stage of infection, which, in turn, enables the transcription of middle and late phage genes with the use of its own RNAP, while the host genome is degraded [42]. Phages belonging to the ϕKMV subgroup (e.g., *Pseudomonas* phage ϕKMV, LKD16, and LKA1) carry an RNAP gene located between the replication and structural module [43]. The localization of the gene encoding KASIA RNAP (*KASIA_p096*) is similar to that of ϕKMV-like phages. Because the *KASIA_p096* gene is located upstream of the structural module, we can assume that it is involved in the transcription of the late KASIA genes. Importantly, T7-like and ϕKMV-like phages belong to the *Autographiviridae* family, while KASIA is classified as a siphovirus. The presence of the RNAP gene in the genome of *Siphoviridae* phages is very rare. Among the siphoviruses, we were able to identify a few phages encoding RNA polymerases (e.g., *Xanthomonas* phage Xp10 [44], Xop411 [45], and OP1 [46]); however, their genes are located in the replication module.

Downstream of the RNA polymerase gene, there is a terminase large subunit (TerL) gene split into three parts by an HNH endonuclease gene (*KASIA_p08*9) (described in Section 2.4). A putative product of the *KASIA_p088* gene shares the highest sequence similarity with TerL of *Colwellia* phage 9A (YP_006489258.1, 64.95% sequence identity), and with numerous putative terminases encoded in the genomes of *Firmicutes*, *Alphaproteobacteria*, and *Archaea,* and acquired from metagenomic analyses. As terminase is a hallmark of tailed viruses, the analysis of the regions containing encoded KASIA_p088 homologous proteins may allow for the identification of potentially complete prophage regions in new groups of bacteria.

In the structural module, we also distinguished 13 genes encoding putative structural proteins: two tail proteins (KASIA_p066 and KASIA_p067), tail assembly protein (KASIA_p068), tail component protein (KASIA_p069), two minor tail proteins (KASIA_p070 and KASIA_p071), tail-tape measure protein (KASIA_p073), a minor tail protein (KASIA_p074), tail protein (KASIA_p076), tail tube protein (KASIA_p077), major capsid protein (KASIA_p082), phage serine protease (KASIA_p084), and portal protein (KASIA_p086). All of them show similarity with a cluster of *Colwellia* phage 9A structural proteins (YP_006489238.1–YP_006489257.1). In order to gain more information about the KASIA structural region, SDS-PAGE and mass spectrometry analyses of the capsid proteins were carried out. This approach allowed for the identification of 13 KASIA-encoded proteins (KASIA_p064–67, _p070–71, p073, p_077–78, p_080, p_082, p084, and p_086) (Figure S3). As a result of this analysis, four previously unannotated hypothetical proteins were described as being virion-associated. Moreover, based on the HHpred searches, the *KASIA_p066* gene seems to encode a polysaccharide depolymerase of which the activity would generate a halo zone around KASIA plaques. The N-terminal end of the KASIA_p066 protein (3–159 aa) is similar to the N-terminal end of the COPG_00052 protein of *Colwellia* phage 9A (YP_006489238.1; 42.59% sequence identity), while the C-terminal end of this protein (180–633 aa) shares similarity with the HYP06_gp005 protein of *Podoviridae* *Vibrio* phage vB_VspP_pVa5 (YP_009876124.1; 43.33% sequence identity) [47]. In phage depolymerases, the N-terminal end provides a connection between the protein and the virion, while the C-terminal domain has enzymatic activity [24].

In the lytic module, we distinguished six genes (*KASIA_p057–062*). The product of the *KASIA_p060* gene contains l-Ala-D-Glu_peptidase-like (cd14845) and peptidase_M15_4 domains (pfam13539), and showed similarity with bacterial metallopeptidases of, e.g., *Vibrio parahaemolyticus* (WP_031856750.1; 60.29% sequence identity) and several phage endolysins of *Salmonella* phages, e.g., phage Shivani (YP_009194885.1; 51.85% sequence identity) [48]. Based on this, we suppose that this gene encodes l-alanyl-D-glutamate peptidase (which hydrolyzes the link between l-Ala and D-Glu residues) and acts as a cell wall lytic enzyme endolysin. It was experimentally demonstrated that the endolysin of the T5 phage with l-alanyl-D-glutamate peptidase activity degrades host peptidoglycans [49]. The other five gene products of the module contain transmembrane helices, which suggests that they may act as a holin and spanins. A BLASTP search in the NCBI virus database revealed no homologs for KASIA_p057–p059 and p061–62 proteins; however, an HHpred search of KASIA_p057 identified a hit to spanin, the outer lipoprotein (99.71% probability).

Additionally, in the KASIA genome, we identified a putative SLT transglycosylase (KASIA_p053), which shared similarity with its counterparts from *Colwellia* phage 9A (YP_006489225.1, 47.80% sequence identity, and YP_006489246.1, 40.91% sequence identity, respectively). Phage SLT transglycosylases are expected to allow entry to and exit from the host cell because they catalyze the cleavage of the glycosidic bonds between N-acetylmuramic acid and N-acetylglucosamine residues in peptidoglycan [50]. The *KASIA_p053* gene is located in the region II encoded proteins potentially involved in nucleotide metabolism, similarly to the *Colwellia* phage 9A transglycosylase gene. Moreover, the KASIA_p053 protein revealed similarity with the proteins of *Shewanella* phage 1/40 (YP_009104197.1; 61.59% sequence identity) and *Shewanella* phage 1/4 (YP_009100506.1; 60.98% sequence identity), of which the genes are also located outside the lytic module, i.e., they are upstream genes encoding replication proteins. Because of the *KASIA_p053* gene localization, it is rather unlikely that its product is involved in the lysis of the host.

The analysis of region IV revealed the presence of at least six genes involved in replication, encoding putative DNA primase (*KASIA_p097*), DNA helicase (*KASIA_p098),* DNA polymerase I (*KASIA_p100*), RNase H (*KASIA_p104*), RuvC resolvase (*KASIA_p105*), and DNA ligase (*KASIA_p115)*. All of these show similarity with their counterparts of *Colwellia* phage 9A. In this region, in both the KASIA and *Colwellia* 9A genomes, two genes encoding ribonucleoside–diphosphate reductase (*KASIA_p108-p109*, and YP_006489268.1 and YP_006489269.1, respectively) and a gene encoding nucleoside triphosphate pyrophosphohydrolase (*KASIA_p111*, YP_006489270.1) are located. The presence of the ribonucleoside–diphosphate reductase (RNR) gene was discovered in 128 phages of the 685 examined genomes (18.7%) [51]. The most common in phage genomes are class I RNR (43%), which are oxygen dependent and only found in aerobic organisms. This class was divided into two subclasses: Ia and Ib [51]. Phages encoding Ia RNR contain both *nrdA* and *nrdB* genes, like the KASIA phage. It was demonstrated that the T4 phage genes *nrdA* and *nrdB* encode the α2 and β2 subunits of ribonucleotide reductase [52]. Bacteriophage T4 RNR appears to be the limiting factor in the initiation and rate of deoxyribonucleotide synthesis in infected cells [53]. In turn, the onset of phage DNA replication is dependent on the turning on of deoxyribonucleotide synthesis [54]. Additionally, in the KASIA replication module, genes encoding oligoribonuclease (*KASIA_p099*), A1 protein (*KASIA_p106*), and deoxynucleoside monophosphate kinase (*KASIA_p112)* were identified. Genes encoding oligoribonuclease were identified in several phage genomes, for example, *Bacillus* phage AR9 [55]. However, the putative product of the *KASIA_p099* gene had no homologues among phage proteins, but showed similarity to the bacterial oligoribonuclease of *Vibrio parahaemolyticus* (WP_082243408.1; 29.70% sequence identity). Oligoribonucleases degrade short RNA oligomers to component 5′-NMPs [56]. HHpred search results showed that the KASIA_112 protein is the most similar to the deoxynucleoside monophosphate kinase of *Escherichia* phage T5 (99.64% probability). It was demonstrated that bacteriophage T5 deoxynucleoside monophosphate kinase catalyzes the phosphorylation of deoxynucleoside monophosphates (dAMP, dGMP, dCMP, and dTMP) to the respective diphosphates using either ATP or dATP as the phosphate donor [57]. Presumably, this enzyme is necessary for the rapid synthesis of substrates for bacteriophage DNA replication in large amounts.

Apart from the above, one of the most interesting genes of the KASIA phage is *KASIA_p106*. Its encoded product is similar to the A1 protein of *Escherichia* phage saus132 (YP_009794839.1; 47.00% sequence identity) [58] and other A1 proteins of T5-like bacteriophages. It was suggested that the A1 protein of the T5 phage may be involved in the degradation of the host DNA and the shutoff of host genes and phage pre-early genes [27]. A1 mutants exhibit a bewildering variety of pleiotropic phenotypes, of which the most obvious is that, in the absence of the A1 gene product, the T5 phage does not inject single-stranded DNA, or cause the degradation of host DNA or the shutoff of host RNA and protein synthesis. Despite the fact that host DNA degradation does not occur in T5 A1 mutants, no nuclease activity has been reported to be associated with the A1 protein [59]. It was also shown that the A1 protein binds to purified host RNAP in infected cells and shuts off pre-early genes [60]. The A1 gene of the T5 phage is located among early genes, together with the A2 protein [27]. However, the location of the *KASIA_p106* gene encoding the putative A1 protein in the replication module is different, and no A2 homolog was found in KASIA. Because of that, the A1 protein of the KASIA phage could not be involved in the degradation of the host DNA, so its function is probably limited to binding and inactivating the host RNAP. So far, only a few phages with the localization of the A1 gene in the replication module, similar to the KASIA phage genome, have been described. PhiCbK gene 126 (*CbK_gp126*) encodes a homolog of the coliphage T5 A1 protein, located transcriptionally slightly upstream of the predicted DNA polymerase gene (*CbK_gp123*). It was postulated that its function may be to alter phiCbK gene expression via interaction with the host RNA polymerase [61].

Within the KASIA replication module, there is also a gene (*KASIA_*p114) encoding a putative type II N6-adenine DNA methyltransferase (m6A MTase). Although the closest homolog of KASIA_p114 is a putative MTase of *Colwellia* phage 9A (YP_006489274.1; 38% identity), it is worth noting that this protein showed considerable similarity in terms of its amino acid sequence to Dam-like proteins encoded by the *Escherichia* viruses T4 (NP_049647.1) and T2 (AYD82627.1), with 27% sequence identity, and to the Dam-like MTase of *Shewanella* sp. M16 (WP_131525404), as well as numerous bacterial DNA m6A MTases of, for example, *Succinivibrio* sp. (MBQ3883869.1) and *Snodgrassella alvi* (RZA00671.1), with 34% sequence identity (Figure S4). By following a previously described procedure [16], we confirmed that GATC sequences are, in fact, substrates for the KASIA_p114 modification protein (Figure S5). Bacterial Dam-like MTases are responsible for a variety of functional roles, including the regulation of DNA replication and segregation, mismatch repair, and the control of gene expression [62]. The intensively studied T4Dam and T2Dam are highly related to their host enzyme, EcoDam [63], in terms of the amino acid sequence and structure [64]. Using a set of mutants, a possible evolutionary pathway between EcoDam and T4Dam was demonstrated [65], but despite the thorough biochemical characterization of T4Dam and T2Dam MTases [66], their role in the viral life cycle is still unclear. It is suggested that their activity most likely ensures the efficient methylation of the phage DNA during the massive viral DNA replication to protect against accidental MutHLS endonuclease cleavage [67]. As the *KASIA_p114* gene is located within the predicted replication module, similarly to the Dam-like MTase genes in T4 and T2 genomes, the relevance of its methyltransferase activity in this stage of the virus reproductive cycle cannot be ruled out.

### 2.4. Analysis of the vB_SspS_KASIA Phage Introns

KASIA carries two HNH endonuclease genes (*KASIA_p090, KASIA_p101*) inserted within the genes encoding the large terminase subunit and DNA polymerase. Closer inspection revealed the presence of group I intron-like sequences, which may have been inserted into the KASIA phage genome through the process of homing [68]. We supposed these introns to contain intervening sequences of TerL and DNA polymerase genes (*KASIA_p088* and *KASIA_p100*). In order to confirm this hypothesis, we isolated total RNA from infected *Shewanella* sp. M16 cells and designed PCR primers binding to sequences of exons flanking putative introns. PCR performed on first-strand cDNA provided smaller products compared to the control reactions on the KASIA DNA genome template (Figure S6). The sequencing of these PCR products revealed the absence of the predicted introns in the cDNA. The self-splicing of these introns completely restores the reading frame of the TerL and DNA polymerase. As was previously mentioned, the products of both these genes are similar to their counterparts in *Colwellia* phage 9A; however, there are no introns in the *Colwellia* phage 9A genome [32]. Group I introns inserted into genes encoding TerL were also found in *Shewanella* phages 1/4 and 1/40 [69]; however, KASIA_p088 and KASIA_p100 proteins share no similarity with them. The model phage containing introns is the *Escherichia col*i T4 phage [34], but group I introns have also been identified in the lysine genes of phages infecting *Streptococcus thermophilis* [70], the thymidylate synthase gene of *Bacillus subtilis* phage B22 [71], and the DNA polymerase genes of *B. subtilis* phages SPO1, SP82, 2C, and phi-e [72]. As in the KASIA phage, they are usually located in important genes encoding enzymes involved in DNA metabolism [73].

### 2.5. Identification and Characterization of Shewanella sp. M16 Prophages

As was mentioned above, the bioinformatics comparison of the M16 draft genome with the nucleotide sequence of the O23S genome [15] showed that they are nearly identical. Therefore, there were three prophage regions in the M16 chromosome, named vB_SspS_MuM16–1 (identical to the mitomycin-induced *Siphoviridae* prophage MuSsp1_O23S), vB_SspM_MuM16–2 (identical to *Myoviridae* MuSsp2_O23S), and vB_SspM_M16–3 (identical to LambdaSsp_O23S). We experimentally confirmed the ability of the MuM16–1 prophage to undergo lysogenic-to-lytic conversion after the exposure of exponentially growing *Shewanella* sp. M16 cells to mitomycin C treatment. The MuM16–1 nucleotide sequence was confirmed by the resequencing of its DNA, which was isolated from purified viral particles. We also identified the presence of variable host sequences at either end of the MuM16–1 genomic DNA, which is probably the result of packaging from the random integration sites used during transpositional replication. The positions, sizes, and putative functions of MuM16–1 encoded proteins are listed in Table S3.

Mitomycin C is a classical chemical agent that can efficiently induce lambdoid prophages. In contrast, Mu-like prophages were known not to be generally inducible by mitomycin C or UV irradiation, or other chemical or physical factors [74]. Experimentally, the induction of lysogenic bacteria was obtained using Mu phages carrying a temperature-sensitive mutation in repressor gene *c* [75]. Elevated growth temperatures considerably increased, as well as RcapMu phage production [76]. Nevertheless, there is an increasing number of reports of mitomycin-induced Mu-like viruses, for example vB_CibM-P1 of *Citromicrobium bathyomarinum* JL354 [77], prophages of *Porphyrobacter* sp.YT40 [78], vB_RhkS_P1 of *Rhodovulum* sp. P5 [79], vB_ThpS-P1 *Thiobacimonas profunda* JLT2016 [80], vB_PeaS-P1 *Pelagibaca abyssi* JLT2014 [80], fF4 *Flavobacterium columnare* ATCC 23,463 [81], and, in this study, MuM16–1 of *Shewanella* sp. M16. We noticed that a putative MuM16–1 repressor C protein (MuM16–1_p01) is analogous to that of bacteriophage lambda, i.e., its amino-terminal region consists of a helix-turn-helix domain (HTH, pfam12844), while its carboxyl-terminal region contains the S24 signal peptidase domain (pfam00717), which is typical for RecA-stimulated autocleavage (Figure S7) [82]. Following this lead, we checked the predicted products of the *c* genes of the abovementioned mitomycin-induced Mu-like phages. In all cases, the overall organization of their repressors was similar to lambdoid bacteriophage repressors, with both S24 family peptidase and DNA-binding HTH domains, whereas the repressors of Mu, D108, and B3 had only HTH domains. On the other hand, the putative BcepMu repressor possesses a S24 peptidase domain, although there are no data on attempts to use mitomycin for its induction, and the *Burkholderia cenocepacia* J2315 prophage, which also possesses the S24 peptidase, was induced at a high temperature [83]. In turn, the SfMu of *Shigella flexneri* lysogens was induced by UV [84], while we were not able to identify the peptidase domain in its repressor but, as in the case of BcepMu, no information on the use of mitomycin C was provided. In conclusion, it seems to us that there is a correlation between the presence of a peptidase domain in the repressor protein and the Mu-like phage sensitivity to induction by mitomycin C, and therefore this is a strong indication to use only this chemical inducer when the S24 peptidase domain is identified in the repressor protein.

In order to verify the MuM16–1 structural proteins predicted by the genomic analysis, its virions were analyzed using mass spectrometry. This approach resulted in the identification of eight MuM16–1 encoded proteins: MuM16–1_p28 (portal protein), p29 (virion morphogenesis), p37 (major head subunit), p42 (tail protein), p43 (tape measure protein), p48 (tail protein), and p45–46, initially annotated as hypothetical proteins, which can now be described as virion-associated proteins (Figure S8).

Inside the structural cluster, there is the most intriguing gene of MuM16–1, i.e., MuM16–1_p33 encoding putative reverse transcriptase (RT). The MuM16–1_p33 protein contains the conserved domain cd03487 (RT_Bac_retron_II, reverse transcriptases in bacterial retrotransposons or retrons; e-value: 1.73 × 10^−61^). Genes encoding similar proteins are present in *Klebsiella* phage ST147-VIM1phi7.1 (YP_009882559.1; 49% sequence identity), *Escherichia* virus P2_2H1 (YP_009877209.1; 46% sequence identity), and *Burkholderia* phage KS5 (YP_004306411.1; 35% sequence identity). In MuM16–1 and the three abovementioned phages, there is a gene encoding putative transcriptional regulator preceding RT genes. MuM16–1_p32 shares 50% of its sequence identity with the YP_009882560.1 of *Klebsiella* phage ST147-VIM1phi7.1, 47% with the YP_009877208.1 of *Escherichia* virus P2_2H1, and 42% with the YP_004306412.1 of *Burkholderia* phage KS5. In each case, the 3′ end of the gene encoding the HTH-containing protein overlaps the 5′ end of the RT gene. The colocalization of this pair of genes allows us to presume that they form a functionally collective genetic element. Additionally, upstream of *Klebsiella* phage ST147-VIM1phi7.1 and the *Escherichia* virus P2_2H1 regulatory genes, there are ORFs encoding the short hypothetical proteins YP_009882558.1 and YP_009877207.1, respectively, which share 78% identity, although their similarity to MuM16–1_p31 is limited (35%). Any of these hypothetical proteins contains an identifiable domain, but only in MuM16–1_p31 did the TMpred program predict the presence of an inner transmembrane segment. We suppose that *MuM16–1_p32–p33* genes comprise a potential retroelement that corresponds to the type VI retron systems [85], where RT genes are associated with DNA-binding protein genes and genes encoding small auxiliary proteins. The location of these putative retroelements in the ST147-VIM1, P2_2H1, and KS5 genomes seems to be conserved at the same loci, i.e., between genes encoding replication and portal proteins, while the MuM16–1 retroelement is localized between genes encoding capsid assembly and morphogenesis proteins. It is also worth noting that these phages are classified in the family *Myoviridae*, in the subfamily *Peduovirinae*, in contrast to siphovirus MuM16–1. Moreover, this putative retroelement seems to be unique to the MuM16–1 phage, as even the analysis of related similar prophage regions in. *S. oneidensis* MR-1 and *Shewanella* sp. LZH-2 did not reveal its presence. Although the surrounding region is highly conserved between these three prophages, neither of the other two carries a similar element. In a recent report, it was demonstrated that retrons function as antiphage defense systems via abortive infection [86]. Further experiments are required to determine whether the Mu16–1 genetic element is also involved in phage exclusion or has some other function.

MuM16–2 and M16–3, like MuSsp2_O23S and LambdaSsp_O23S of *Shewanella* sp. O23S, seem to be inactive prophage elements. We obtained the sequences of the MuM16–2 and M16–3 genomes during the analysis of the *Shewanella* sp. M16 genome assembly. Although MuM16–2 and M16–3 were split across multiple contigs, we confirmed their genome sequence by PCR, with primers binding to the 3′ end of one contig and the 5′ end of a potentially adjacent one, and the Sanger sequencing of those products. We suppose that M16–3 was not capable of forming infectious particles, probably due to a point mutation in its integrase gene (transition G to A in the tryptophan codon TGG, resulting in the stop codon TAG), which probably split the initial gene into two ORFs. The positions, sizes, and putative functions of MuM16–2 and M16–3 encoded proteins are listed in Tables S4 and S5, respectively.

### 2.6. Comparative Genomic Analyses

The annotation process revealed that the KASIA phage showed the highest similarity to *Colwellia* phage 9A, both in terms of its genome organization and the sequences of the encoded proteins (see above). Nevertheless, we sought to investigate how it compares to other known phages deposited in NCBI databases. We therefore performed a sensitive search of all of the proteins encoded by the KASIA phage against a set of proteins from 14,470 phages using phmmer (e-value 1 × 10^−5^ threshold) [87]. As a result, we observed that 4476 phages (including *Colwellia* phage 9A) encoded at least one protein that showed similarity to one of the KASIA proteins (Figure S9, Table S6). In total, the *Colwellia* phage 9A encodes 49 similar proteins; other phages encode at most 14 such proteins, which suggests the lack of a significant phylogenetic relationship between KASIA and the other analyzed phages, except 9A. Moreover, only the 9A shared homologs were encoded in a collinear cluster of genes (i.e., the structure and morphogenesis module). On the other hand, the predicted products of KASIA genes involved in nucleotide metabolism and replication had the greatest number of homologs among the proteins encoded by the known viruses. The most numerous were homologs of KASIA_p108 and _p109, which were predicted as alpha and beta subunits of ribonucleotide reductase (Figure S10, Table S6). Moreover, all of the phages encoding at least 10 homologous proteins (66 phages) had genomes exclusively larger than KASIA phage one, i.e., ranging from 101,637 bp (*Vibrio* phage 2.058.O._10N.286.46.B8, MG592662) to 349,331 bp (*Vibrio* phage V07, MT135025), and there were only *Myoviridae* phages, except for *Colwellia* phage 9A (*Siphoviridae*).

**Figure 3:**
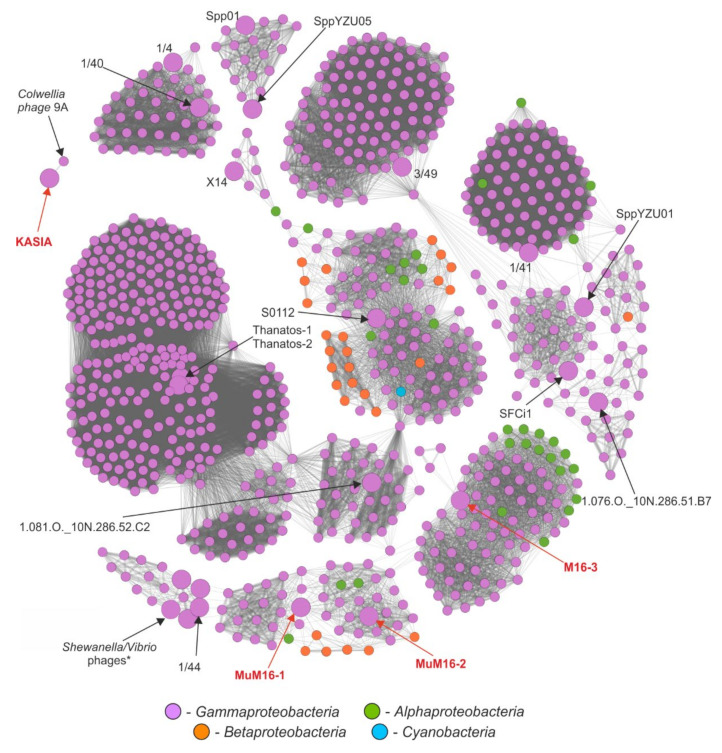
Protein-based phage similarity network. Only *Shewanella*-infecting phages and their neighborhood, i.e., phages showing significant similarity and connected with them via edges, are shown. Nodes, each representing a phage, corresponding to *Shewanella* phages, are enlarged and indicated with arrows, except for the *Shewanella*/*Vibrio* phages, which are indicated as a group of four phages. The coloring of the nodes is based on the taxonomic class of the phage hosts. The edge thickness reflects the degree of similarity between two phages (the greater the similarity, the thicker the edge), considered as an overall similarity of proteins encoded by a pair of phages.

In order to better understand the diversity and relationships among phages infecting *Shewanella* spp., protein-sharing networks were generated with vConTACT v2 [88]. For this analysis, we also included three complete *Shewanella* sp. M16 prophages. From the 14,470 phage-predicted proteomes, we extracted a subnetwork of *Shewanella*-infecting phages and their relatives. These included the KASIA phage, three prophages from the M16 strain, and other *Shewanella*-infecting phages, as well as a set of viruses connected with them via edges. This resulted in a final network of 23 *Shewanella* (pro)phages and 892 other phages, connected by 38,709 edges in total (Figure 3 and Figure S11).

The results of the networking showed how diverse the known *Shewanella* phages are. They are scattered across 13 different clusters, suggesting a high diversity of phages infecting these bacteria that is yet to be discovered. Of the 23, two Thanatos, Spp01 and YZU05, 1/4 and 1/40, as well as 1/44 and *Vibrio*/*Shewanella* phages (four out of the six viruses of the Nahant Collection [19] obtained using *Shewanella* spp. as hosts, as mentioned in the introduction, but placed in the GenBank database under names suggesting that the host belonged to *Vibrio* genus), reflected high identity clustering within the same cliques and vConTACT viral clusters. Our newly isolated KASIA phage, as expected, clustered exclusively with *Colwellia* phage 9A. Both phages were classified as representatives of a single viral cluster, but as two separate viral subclusters (Table S7). The fact that both of them acted as outliers within the whole network emphasizes their uniqueness; while *Colwellia* phage 9A is considered the only representative of the *Franklinbayvirus* genus (according to ICTV Virus Metadata Repository (version 18 May 2021; MSL36)), the KASIA phage could be considered the first of a novel genus with the proposed name *Goldenslopevirus*.

Despite the variety, the *Shewanella* phages of which the genomes are available in public databases mostly showed similarities to viruses infecting other genera of *Gammaproteobacteria*; these constituted 862 out of the 915 presented phages. Of the phages infecting other classes of bacteria, 21 were *Alphaproteobacteria*, 31 were *Betaproteobacteria*, and one was from the *Cyanobacteria* (Figure 3 and Figure S11a). At the genus level, the dominant phages were those infecting *Escherichia* (267 phages), *Vibrio* (129), *Pseudomonas* (109), *Salmonella* (92), *Klebsiella* (50), and *Aeromonas* (30), which in total constituted 74% of all of the presented phages (Figure S11b).

**Figure 4:**
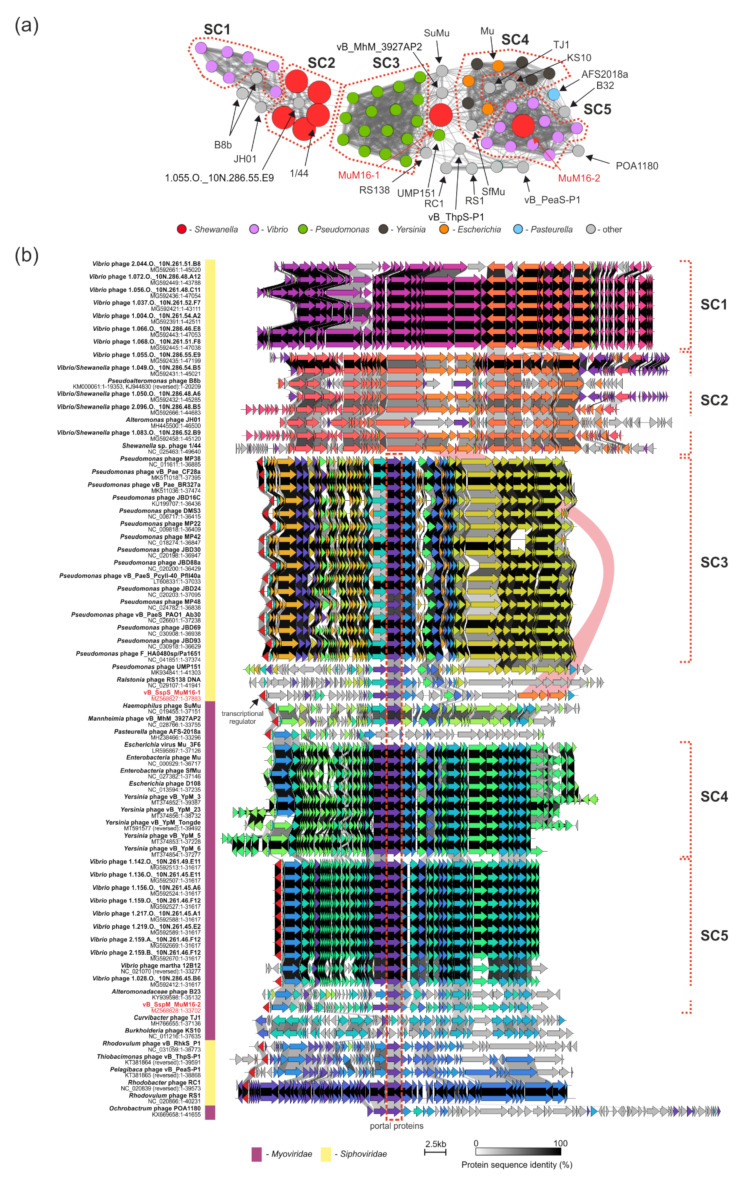
(**a**) Genomic comparison of the Mu-like phages’ similarity network neighborhood and (**b**) the alignment of these phage genomes. In (**a**), subclusters of phages grouped in the same viral clusters by vConTACT are marked with a dashed red line. Phage genomes in (**b**) are delineated and reversed if required. Each protein-encoding feature is presented as an arrow. The two arrows connected with a white-to-black colored block correspond to their protein sequence identity level. Subclusters corresponding to those from (**a**) are also marked. The location of most of the portal proteins used for genome alignment is marked with a red dashed rectangle.

The majority of the clusters were composed of phages belonging exclusively to one phage family: *Myoviridae*, *Siphoviridae, Chaseviridae*, or *Podoviridae* (Figure S11c). These were further divided into lower taxonomic ranks (Figure S11d). However, there were phages encoding proteins which are similar to more than one phage family, acting as so-called bridges between two groups of phages. One of the clusters included three *Shewanella* phages: 1/44, MuM16–1, and MuM16–2. Within this particular cluster, one can distinguish five subclusters (SC1–5) of more closely related phages (a greater thickness of edges connecting the nodes) and several outliers, which are also partially reflected by the fact of infecting the same genus of bacteria, e.g., phages infecting *Vibrio* (magenta) and *Pseudomonas* (green) (Figure 4a). The SC1 and SC2 subclusters were composed of *Siphoviridae* phages. Among them, there were *Shewanella* 1/44, *Alteromonas* JH01, *Pseudoalteromonas* B8b, and viruses isolated from environmental samples (the Nahant Collection), with *Vibrio* and *Shewanella* strains as hosts [19]. Those from SC2—i.e., *Shewanella* phage 1/44, *Vibrio* phage 1.055.O._10N.286.55.E9, and *Shewanella/Vibrio* phages 1.049.O._10N.286.54.B5, 1.050.O._10N.286.48.A6, 1.083.O._10N.286.52.B9, and 2.096.O._10N.286.48.B5—encoded proteins that were similar to the structural proteins of the MuM16–1 prophage (Figure 4b). In particular, there were five (out of 17) structural protein products of adjacent genes of that phage, i.e., p45–p49. The mass spectrometry analysis of the MuM16–1 virions (see above) revealed that p45, p46, and p48 were present in its particles, possibly as parts of tails. Additionally, the applied machine-learning-based classification of these structural proteins using PhANNs [89] allowed us to hypothesize that p47 is a head–tail-joining protein (81% confidence), while p48 is a shaft or tail tube protein (100% confidence). Because MuM16–1 is a siphovirus, and because we observed a conserved synteny of structural genes among SC2 members infecting *Shewanella* strains, we hypothesize that the whole set of five conserved genes might be involved in the recognition of the bacterial surface receptors required for the successful infection of a cell. It is also worth noting that the products of these five genes are, in fact, the only ones that allow for connecting Mu-like phages with the SC2 and, through it, also the SC1 subclusters of non-Mu-like *Siphoviridae* phages. MuM16–1 acts here as a so-called “bridge” between these two groups of viruses.

Within the Mu-like phage clusters, MuM16–1 is located between SC3 (composed of representatives of the genus *Casadabanvirus* infecting exclusively *Pseudomonas* spp.), SC4 (*Muvirus*), SC5 (unclassified Mu-like viruses), and a group of *Alphaproteobacteria* phages (Figure 4a). Together with *Mannheimia* vB_MhM_3927AP2 and *Haemophilus* SuMu phages, they act as a bridge connecting subclusters SC3–SC5. We investigated the diversity of Mu-like phages and the reasons for the MuM16–1 clustering location by comparing the phages with Clinker [90]. We observed highly conserved genome organization both within and between the subclusters (Figure 4b). All of the Mu-like phages, except for *Curvibacter* TJ1, *Burkholderia* KS10 (two out of 3 *Betaproteobacteria*-infecting phages), and *Pasteurella* AFS-2018a, were aligned based on the location of the gene encoding a portal protein (Figure 4b); it was previously discovered that portal and terminase are two of the most conserved proteins among phages due to their role in packaging phage DNA into capsids [91]. A distinctive feature of Mu-like phages is their unique way of packaging their DNA into a capsid directly from a host genome. The alignment also showed that MuM16–1 demonstrated similarity to other Mu-like phages through 12 out of 52 proteins. These were phage repressor protein CI (p01), regulatory protein Gem A and the regulator of late transcription protein C (p12–13), transcriptional regulator (p24), small and large terminase subunits (p26–27), portal protein (p28), two phage virion morphogenesis proteins (p29–30), protease and major head subunit proteins (p36–37), DNA adenine methylase (p50), and two hypothetical proteins (p09 and p40). These seemed to be conserved among both *Siphoviridae* and *Myoviridae* Mu-like phages, which we verified by generating a protein-based similarity network of the analyzed Mu-like phages’ predicted proteomes (Figure S12). This analysis confirmed the observation, as all of the proteins listed above were located within clusters composed of proteins encoded by both *Siphoviridae* and *Myoviridae* Mu-like phages. In particular, portal and virion morphogenesis (Mup31-like) proteins created the most numerous clusters, composed of 51 proteins each, while less-numerous clusters were composed of Mup36-like hypothetical proteins (encoded by 47 phages), proteases (46), major head subunits (46), and transposases (43). The other clusters had at most 33 proteins (CI repressor) (Table S8). In reference to a previous paper on portal and terminase proteins [92], both the small and large subunits were found to fall into clusters of 30 proteins, which indicates the higher diversity of these proteins and the potential superior role of the portal protein in a DNA packaging mechanism specific to Mu-like phages. Moreover, this analysis showed that portal, Mup36-like, two virion morphogenesis, protease, major head subunit, and transposase proteins, supplemented with Mu-like CI repressor, Mor and late transcription C, Gam, GemA, Ner, Mup26-like, and terminase subunits proteins, could be considered Mu-like phage hallmark proteins.

In contrast to MuM16–1, close relatives of MuM16–2 have already been discovered in environmental samples of ocean coastal water. MuM16–2 shows high similarity to phages from the SC5 subcluster, which is exclusively composed of another set of *Vibrio* phages [19] and a *Vibrio* Martha 12B12 phage from an Atlantic water sample. All of these were also classified as members of a single viral cluster (Table S7). It is, therefore, another example of a phage infecting a strain isolated from a gold mine, which has significant similarity to phages isolated from distant geographic locations.

The last of the M16 prophages, M16–3, was located outside of a large cluster of the versatile *Peduovirinae* subfamily. It acted as a bridge connecting them to three *Vibrio* phages, i.e., vB_VpaM_MAR, VP585, and VHML. Within that cluster there are *P2viruses* to which we observed similarity at both the gene organization and protein levels during the M16–3 genome annotation.

### 2.7. Functional Characterization of vB_SspS_KASIA

#### 2.7.1. Sensitivity to Temperature of the KASIA Phage

**Figure 5:**
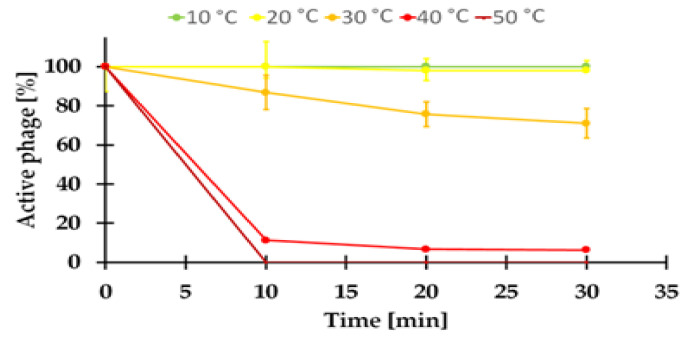
Phage KASIA stability under various temperatures.

The viability of the KASIA phage at a temperature range of 10–50 °C was investigated. The virus was sensitive to temperatures ≥ 40 °C, with an immediate loss of infectivity at 50 °C (Figure 5). A similarly limited tolerance to higher temperatures was previously demonstrated for the temperate *Serratia* phage BZS1 isolated from the same environment, but from rock biofilms [16]. However, the stable low temperature in this gold mine (10–11 °C) [9,10] is probably sufficient for the propagation and survival of these viruses.

#### 2.7.2. The Influence of Various Conditions on the KASIA Phage Plaque Formation

The KASIA phage was originally isolated with the use of a spot assay (see Section 3.2) on the M16 strain cultivated on LB medium at 20 °C (selected as being optimal for the growth of M16, Figure S1), but the obtained plaques were poorly visible. As the plaque yield is affected by physicochemical factors (e.g., pH, ions, temperature [93]), we used the double agar overlay technique to check whether a change in medium and temperature would improve the visibility of the plaques. Therefore, the plates (LB and nutritionally reduced R2A medium) were incubated at 4 °C, 10 °C, 20 °C, and 30 °C, and were examined after 24, 48, 72, and 120 h. We observed that the KASIA phage formed clear plaques only when the M16 strain was cultivated on R2A medium at 4–20 °C. The time of the plaque appearance was dependent on the incubation temperature of the plates (which, in turn, was correlated with the bacterial lawn appearance): at 20 °C, clear plaques were visible after 24 h; at 10 °C, after 48 h; and at 4 °C after 72 h, although, at 20 °C, the plaques were smaller than at 4–10 °C (Figures S13 and S14). In each case, growing turbid halos around plaques appeared approximately 24 h after the plaque formation. On the M16 lawn obtained on LB medium, the poor visibility of single plaques was not improved even at 4–10 °C; nonetheless, lysis zones were generated after the application of 10-µL drops of the high titer KASIA suspension (more visible at ≤10 °C; see Figure S15). The obtained results emphasize that the optimum conditions for phage production (4–10 °C) do not necessarily correspond to those for bacterial growth (at 20–30 °C and in LB medium, the strain grows three times faster than in the R2A medium; see Figure S1). A similar observation, i.e., of a more restricted temperature range for the virus than for the host, was made for the extreme cold-active 9A phage of *Colwellia psychrerythraea* 34H. In this case, this phenomenon is explained by the extreme thermolability of the 9A [6].

#### 2.7.3. The Impact of the Medium Type on the KASIA Phage Adsorption Rate

**Figure 6:**
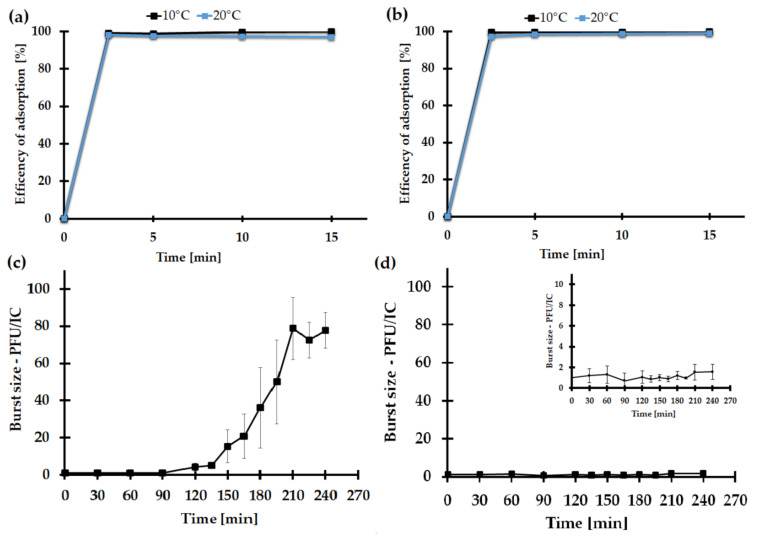
Phage KASIA development. (**a**) The adsorption of the phage particles to the bacterial cells was assessed after 1, 2.5, 5, 10, and 15 min in R2A medium and (**b**) LB medium. (**c**) One-step growth curve in R2A medium and (**d**) in LB medium, conducted for 240 min and assessed in 15–30-min time intervals. The graph for the LB medium is presented in two versions: on the same scale as R2A (to illustrate the difference in the number of phage particles produced) and on a scale 10 times smaller (for the better visualization of the results obtained on the LB medium). Each experiment was conducted five times, and the average values are plotted with standard deviation values.

As LB is a nutritionally rich medium, and because its components (or the products of bacterial metabolism in this medium) could potentially limit the adsorption efficiency of the phage, we studied the kinetics of KASIA adsorption to *Shewanella* sp. M16 cells in LB and R2A media. This revealed KASIA phage rapid cell adsorption in both media, at 10 °C and 20 °C. After 2.5 min incubation, approximately 99% of the phages were attached to the host cells, and the attachment efficiency of KASIA was stable during the whole experiment (Figure 6a,b). Therefore, we concluded that there were probably no factors in the LB medium that interfered with phage adsorption to M16 cells.

#### 2.7.4. One-Step Growth of the KASIA Phage

Besides the phage’s adsorption to the bacterial cells, the plaque productivity depends on the efficiency of the virus propagation in the host cell. Therefore, in order to explore the KASIA development in *Shewanella* sp. M16 cells, a one-step growth assay in LB and R2A media at 10 °C was employed. It should be emphasized that, although the one-step growth analysis was conducted separately in LB and R2A liquid media, the plaque assay (and then the estimation of the phage titer) for both experiments was conducted on R2A double agar plates. Experiments carried out in R2A revealed that the latency period of KASIA was about 120 min, and the rise period was 210 min. The burst size of the KASIA phage in this medium was 80 ± 17 PFU/IC (Figure 6c). Surprisingly, in the one-step growth analysis in the LB medium, no change in the PFU KASIA phage was observed over the examined time (Figure 6d).

Most studies of bacteriophage development and growth are performed under optimal conditions for the host cell but, as is widely known, these conditions may not be optimal for the phage [94]. It seems that the KASIA phage is a good example of this. While *Shewanella* sp. M16 grows faster in LB medium at 20–30 °C (Figure S1), the KASIA phage yield is negligible in these conditions. This is probably not the result of the potential thermolability of the KASIA proteins, as viral particles seem to be relatively stable at this temperature (Figure 5), nor of the presence of chemical compounds in the LB medium limiting the viral particles’ access to the cell surface, because we did not observe any differences in the efficiency of the KASIA phage adsorption in LB and R2A media (Figure 6a,b).

In order to determine whether the genetic information of the KASIA phage is expressed at all in infected M16 cells cultivated in LB medium, we decided to check for the presence of viral RNA after infection. We took advantage of the fact that the mRNA of *KASIA_p088* and *KASIA_p100* (putative terminase and DNA polymerase, respectively) would be shorter than the full-length segments comprising these genes due to the presence of introns. Therefore, we used the same procedure (Section 2.4) to demonstrate that the introns present in *KASIA_p088* and *KASIA_p100* were absent in the transcripts. The only modification in this experiment was the medium for the M16 culturing—LB instead of R2A. RT-PCR products derived from cDNA that were smaller than those obtained from KASIA genomic DNA were clearly detected, indicating that mature spliced terminase and DNA polymerase mRNAs were produced.

As the KASIA phage productivity seems much greater when its host is cultivated on a nutritionally reduced medium, it cannot be ruled out that an antiviral protection system of M16 is activated (or works more efficiently) in LB. In the case of, e.g., a functional abortive infection system, the induction of such a defense strategy would kill the infected cells, stopping phage propagation. A putative abortive infection system has been identified in the MuM16–1 prophage retroelement (see above); however, the correlation of the low efficiency of the phage multiplication in the LB medium with the involvement of a host defense mechanism requires further research. It is worth stressing that, in the stable and oligotrophic environments of the gold mine, the host of KASIA does not in fact encounter such favorable growth conditions (e.g., a rich medium or temperature ≥ 20 °C) to trigger the potential antiphage defense system; hence, the virus does not encounter selection pressure that would result in the evolution of a counteraction system.

However, the explanation may be even simpler. As viral replication takes advantage of the host’s metabolism, it is possible that, under artificially created conditions (≥20 °C, LB medium) distant from those in the gold mine, the host’s metabolic state does not match the virus’s need for efficient replication and, as a result, the host becomes resistant to the phage.

#### 2.7.5. Host Range

The host range of the phage KASIA was tested on seven environmental strains of *Shewanella* spp. and other strains (e.g., *Serratia* spp. and *Aeromonas* spp.) isolated from the Zloty Stok mine by a spot test of the KASIA phage lysate. The bacterial strains used in this assay are listed in Table S9. All of the *Shewanella* spp. isolates from the Zloty Stok mine, including *Shewanella* sp. O23S, were susceptible to infection by KASIA. No plaques were obtained with any of the other strains tested. *Shewanella oneidensis* MR-1 and *Shewanella baltica* OS155 were also insensitive to the KASIA phage. This might suggest that KASIA has a narrow host range, possibly confined to the *Shewanella* spp. strains inhabiting the microbial mats of the Zloty Stok mine.

## 3. Materials and Methods

### 3.1. Bacterial Strains, Plasmids, and Culture Conditions

The bacterial host of the KASIA phage *Shewanella* sp. M16 was isolated from microbial mats of the Zloty Stok gold and arsenic mine (SW Poland) collected in September 2018. A sample of the microbial mats was incubated with 50 mL of LB medium at 10 °C. After 7 days, the suspensions were centrifuged, and the supernatant was diluted and plated on LB agar. After obtaining a pure culture, the genomic DNA was isolated and 16S rRNA analysis was conducted. Using the same methodology, other *Shewanella* spp. strains (M15, ZGL8, ZGL10, and ZGL11) were isolated from other samples of mats. They were used in a host range testing assay, together with various strains isolated from the same environment and other *Shewanella* strains (*S. oneidensis* MR-1 and *Shewanella baltica* OS155). The bacteria used in the host range testing (Table S9) were grown under aerobic conditions on double-layer R2A agar plates at 20 °C, except for *S. baltica* OS155, which was cultivated on solid Marine broth medium (MBM).

The following *Escherichia coli* strains were used in this study: TOP10F’ (Invitrogen, Waltham, MA,USA), ER2566 (New England BioLabs, Ipswich, MA, USA), and ER2929 Dam- strain lysogenized with DE3 element, which carried the gene for T7 RNA polymerase under the control of the lacUV5 promoter [95]. These were cultured under the standard conditions in LB medium at 37 °C. When required, the media were supplemented with kanamycin at 50 µg/mL and ampicillin at 100 µg/mL. The plasmids pUC19 (Thermo Fisher Scientific, Waltham, MA, USA) and pET30a (Invitrogen, Waltham, MA, USA) were used as cloning or expression vectors, respectively.

### 3.2. Isolation of the KASIA Phage

The phage KASIA was isolated from microbial mats of the Zloty Stok mine collected at the same time (September 2018) as the *Shewanella* sp. M16 strain. Samples of the mats, together with mine water, were centrifuged (14,000× *g*, 30 min) to remove the solid impurities. The supernatants were filtered through a 3-µm pore-size, low-protein-binding membrane filter (MerckMillipore, Darmstadt, Germany) and a 0.22 µm pore-size membrane filter (MerckMillipore, Darmstadt, Germany) to remove microorganism cells. Next, 10 µL of the processed environmental sample was spotted on the top layer of 0.5% LB agar medium containing 100 µL of the bacterial host *Shewanella* sp. M16 spread on solid LB to create a bacterial lawn. The plates were incubated at 20 °C for 24–48 h, and then were inspected for lysis zones at the sites of the spotting. The pure culture of the bacteriophage was obtained after three single-plaque purifications using the double-layer agar plate method using R2A medium and *Shewanella* sp. M16 host bacteria. Plaques picked from the third subculture were used to produce the high-titer KASIA phage stock by using the confluent plate lysate method on R2A medium. Saline magnesium (SM) buffer (100 mM NaCl, 50 mM of Tris Cl pH 7.5, 8 mM of MgSO_4_ × 7H_2_O)) was used for the long-term storage of the phage suspension at 4 °C, and for the phage dilution.

### 3.3. Transmission Electron Microscopy (TEM)

The TEM analysis was conducted as described previously [16]. The visualization of the phages was performed at the Core Facility of the International Institute of Molecular and Cell Biology (IIMCB, Warsaw, Poland).

### 3.4. DNA Isolation and Sequencing

The total DNA of *Shewanella* sp. M16 was extracted from the overnight culture carried out in the LB medium with a Genomic Mini kit (A&A Biotechnology, Gdansk, Poland). The whole-genome shotgun sequencing of the M16 strain was conducted by Biobank Lab, University of Lodz (Lodz, Poland) on an Illumina MiSeq platform at a read length of 2 × 250 bp. The MuM16–1 prophage was induced by mitomycin C (MilliporeSigma, Darmstadt, Germany). The bacterial culture was grown in LB medium to optical density at 600 nm (OD600) of 0.3. The culture was then treated with mitomycin C (500 ng/mL), and its growth (with shaking) was continued for 18 h. The KASIA phage and MuM16–1 prophage total DNAs were isolated by phenol–chloroform extraction and isopropanol precipitation [96]. The phage genome sequencing was performed by the DNA Sequencing and Oligonucleotide Synthesis Laboratory (oligo.pl) (Institute of Biochemistry and Biophysics, Polish Academy of Sciences, Warsaw, Poland), also on the Illumina MiSeq platform, but at a read length of 2 × 300 bp. The raw reads acquired from the above sequencing projects were subjected to a quality check and filtering with the application of FastQC v.0.11.5 [97] and fastp v.0.20.0 [98]. During the fastp run, the following parameters were applied: --detect_adapter_for_pe --cut_window_size 6 --cut_tail --cut_mean_quality 19 --length_required 50 --n_base_limit 5 --trim_poly_x --poly_x_min_len 10 --correction --overlap_len_require 20 --overlap_diff_limit 5 --trim_front1 13 --trim_front2 *13*. The filtered reads were then assembled with SPAdes v.3.15.0 in a careful mode [99]. The analysis of the genomes’ sequence coverage, including the analysis of the redundant regions, was performed by mapping the filtered reads against the assemblies with bwa mem v.0.7.17-r1198-dirty [100] and samtools v.1.10 [101]. Then, the alignments were viewed in Integrative Genome Viewer v.2.6.2 [102]. Additional analysis of the phage termini and packaging mechanisms was conducted with the application of PhageTerm v.1.0.12 using the filtered reads as the input [26].

### 3.5. Genome Annotation

The bioinformatics characterization of the nucleotide sequence of the phages was performed using Clone Manager 8 (Sci-Ed, Westminster, CO, USA) and Artemis v.16.0.0 software [103]. The genomes were automatically annotated using the RASTtk [104] in phage mode on the PATRIC website [105], and the annotations were then precisely manually vivificated using BLASTP, UniProt, Pfam and HHpred (PDB_mmCIF70_11_Oct, SCOPe70_2.07, 794 COG_KOG_v.1.0, Pfam-Av33.1, and NCBI_Conserved_Domains (CD)_v3.16 or the nr50_1_Oct, 795 databases) [106] algorithms for the similarity searches. Putative tRNA genes were identified using the tRNAScan-SE [107] and ARAGORN programs [108]. A phage family search was carried out using VIRFAM [109].

### 3.6. Comparative Analysis

The genomes of *Shewanella* sp. M16 and *Shewanella* sp. O23S were compared with the application of blastn and circoletto [110]. The genomes of the KASIA phage and *Collwelia* phage 9A were also compared using circoletto, but were supplemented with the blastp output of the comparison of proteins they encode. During the search, the following thresholds were applied: an *e*-value of 1 × 10^−5^ and a minimum query coverage of 75%. The genomes of KASIA, MuM16–1, MuM16–2, M16–3 phages were compared with other known phage genomes recovered from the RefSeq (if possible) and GenBank databases based on the summary provided by MillardLab as of February, 2021 (http://millardlab.org/bioinformatics/bacteriophage-genomes/phage-genomes-feb2021/ (accessed on 8 August 2021)). Redundant records referring to the same genomes but originating from the RefSeq and GenBank databases were removed, and only the RefSeq record was retained. Their proteins were used for the search against a set of proteins encoded by the KASIA phage with a phmmer run with an e-value of 1 × 10 ^−5^ as the threshold [87]. Moreover, all of the phage genomes extended with the phage genomes analyzed in this study were subjected to the overall genome comparison analysis by vConTACT v0.9.20 [88], with a ProkaryoticViralRefSeq99-Merged dataset as a reference. The input records overlapping with the ones from the reference dataset were removed from the analysis to avoid redundancy. During the program’s run, MCL and ClusterOne algorithms were applied for the protein and viral clusters’ inflation, respectively. The other parameters were left default. The resulting network was further filtered with a Python script to retain a subnetwork of nodes representing *Shewanella*-infecting phages and their neighborhood, i.e., other nodes they were connected to. The additional comparison of the selected phage genomes was performed with the application of clinker using the default settings [90]. The supplemented protein similarity network was generated based on the comparison of protein sequences using an all-against-all blastp search with the following thresholds: an *e*-value of 1 × 10^−5^ and 85% sequence coverage per HSP. The results were then parsed with a Python script to generate a network file in which the edge weight was calculated by multiplying the query coverage and percent identity of each of the two proteins. All of the analyzed networks were visualized with Gephi v.0.9.2 [111], and the nodes were laid out in two-dimensional space with the application of Fruchterman-Reingold [112], ForceAtlas 2 [111] and Noverlap algorithms.

### 3.7. SDS-PAGE and Mass Spectrometry Protein Analysis

The SDS-PAGE and mass spectrometry protein analysis were conducted as described previously [16]. The mass spectrometry analysis was performed in the Mass Spectrometry Laboratory, Institute of Biochemistry and Biophysics, Polish Academy of Sciences (IBB PAS) (Warsaw, Poland).

### 3.8. RNA Isolation and In Vivo Splicing Assay

An overnight culture of *Shewanella* sp. M16 (R2A or LB medium, at 20 °C) was refreshed and grown in the same conditions until OD_600_ = 0.2, and then infected with the KASIA phage at an MOI of 1. After 10 min, 3 mL of the culture was centrifuged, suspended in fenzol (A&A Biotechnology, Gdansk, Poland), and stored at 4 °C for 24 h. The total RNA was isolated using an Total RNA Mini kit (A&A Biotechnology, Gdansk, Poland). The isolated RNA was additionally purified with TURBO Dnase (Thermo Fisher Scientific, Waltham, MA, USA) and reverse transcribed using KASIA_Ter5 and KASIA_Pol1 primers. The cDNA was amplified by PCR with Phusion Polymerase (Thermo Fisher Scientific, Waltham, MA, USA) and pairs of primers: KASIA_Ter1–2, KASIA_Ter3–5, KASIA_Pol1–2 and KASIA_Pol3–5, respectively (Table S10).

### 3.9. Cloning, Overexpression, Purification, and Testing the Specificity of a Putative DNA MTase

The DNA encoding KASIAp114 was amplified by PCR, using specific primers (Table S10) designed to incorporate the NdeI and XhoI sites at the 5′ and 3′ ends of these genes, respectively. The amplified DNA fragment was cut with NdeI and XhoI, and cloned into the NdeI/XhoI-digested pET30a, yielding the pET_KASIAp114 construct. The protein expression and restriction enzyme digestion protection assay for revealing the sequence specificity were carried out as previously described [113].

### 3.10. Confirmation of the M16–3 Genome Assembly

The genomic DNA of *Shewanella* sp. M16 was used as a template in PCR with primers: M16_3_1 and M16_3_2, M16_3_3, and M16_3_4, M16_3_5 and M16_3_6, respectively (Supplementary Table S10). The amplified DNA fragments were cloned into SmaI-predigested pUC19 plasmid, and sequenced.

### 3.11. Determination of the Phage Host Range by Spot Testing

The phage host range was determined by adding 5μL KASIA phage suspension dropwise onto the surface of double-layer R2A or MBM agar plates inoculated with the appropriate bacterial strain.

### 3.12. Thermal Stability of the Phage

For thermal stability, the phage lysate (10^10^ PFU/mL) was diluted 100 times in R2A medium and incubated at 20, 30, 40, and 50 °C. The control sample was incubated at 10 °C. After 10, 20, or 30 min, the numbers of PFU were determined by the serial dilution of phage mixtures and a plaque assay on R2A double agar plates with *Shewanella* sp. M16 as the host. The plates were incubated for 48 h at 10 °C.

### 3.13. Adsorption Kinetics

In total, 10 µL KASIA phage (10^10^ PFU/mL) was mixed with 1 mL *Shewanella* sp. M16 overnight culture and incubated in LB and R2A medium, without shaking, at 10 and 20 °C. After 2.5, 5, 10, and 15 min, samples were collected and centrifuged to remove the phage-adsorbed cells. The supernatant was used in a plaque assay for the determination of the unadsorbed phage titer. The plates were incubated for 48 h at 10 °C. The number of adsorbed phages was determined based on the ratio between the starting concentration of phages added and the concentration of the unadsorbed phages. The data were obtained from three independent experiments.

### 3.14. One-Step Growth Curve

After reaching OD600 = 0.2 (18–20h in R2A medium), the *Shewanella* sp. M16 liquid culture cultivated in LB or R2A was mixed with the KASIA phage to obtain an MOI of 0.0001, and incubated without shaking at 10 °C. After 15 min of adsorption, the mixtures were centrifuged to remove unadsorbed phage particles, and then suspended in fresh LB or R2A medium. The number of infection centres (ICs) was determined as the numbers of PFU/mL at the beginning of the experiment. The experiment was further carried out at 10 °C with shaking (150 rpm). At the appropriate time points, the numbers of PFU/mL were determined by the serial dilution of the suspension collected from the reaction flask and a standard plaque assay on R2A double agar plates. The plates were incubated for 48 h at 10 °C. The burst size was presented as PFU/IC. The data were obtained from five independent experiments.

### 3.15. Nucleotide Sequence Accession Numbers

The complete nucleotide sequence of the *Shewanella* sp. M16 strain genome, as well as the KASIA, MuM16–1, MuM16–2 and M16–3 phage genomes, can be accessed under the following GenBank accession numbers: NZ_JAGTUL010000000, MZ568826, MZ568827, MZ568828, and MZ568829.

## 4. Conclusions

In this study, we described a novel phage–host system—vB_SspS_KASIA and *Shewanella* sp. M16 (which harbors three prophages: mitomycin-inducible vB_SspS_MuM16–1, vB_SspM_MuM16–2 and vB_SspM_M16–3).

The KASIA phage can be defined as cold-active, as it is able to produce plaques at 4–20 °C; however, the visibility of the formed plaques dropped at temperatures ≥ 20 °C. Moreover, clear plaques were only visible on R2A, and we were able to demonstrate efficient viral particle production only in this medium. At the same time, we showed that its host, *Shewanella* sp. M16, grows faster in a nutritionally richer LB medium at 20–30 °C. Thus, we presume that the KASIA phage would not be able to propagate outside of its natural habitat of the gold mine, in contrast to its host. We suppose that the KASIA phage and the M16 strain might be an attractive model system for studying the impact of the environment on virus–host dynamics and the ecological consequences of the potentially greater flexibility of the M16 strain to live in a different ecological niche than its parasite. Furthermore, to the best of our knowledge, there are only a few described bacterial viruses isolated from an environment with such multiple extremes as the Zloty Stok gold mine, making the KASIA phage an interesting target for further physiological analyses. Our results confirm the importance of acquiring data and studying phage–host dynamics under experimental conditions mimicking those of the tested environment as closely as possible. Moreover, they suggest that, without screening many conditions of host cells’ culture, some bacteriophages might be undetected or at least cannot be propagated in a laboratory.

Through this study, we showed that the KASIA phage has the largest genome among the siphoviruses infecting representatives of the *Shewanella* genus. The length of its genome (about 91 kb) is quite rare in known bacterial viruses, as are its 6912 bp-long nonpermuted direct terminal repeats. Having the smallest genome among myo- and siphoviruses with fixed terminal repeats, the KASIA phage could be convenient for studying this still unknown mechanism of generating such ends. Moreover, it cannot be ruled out that the genome transfer mechanism of the KASIA phage is different from that of the intensively studied T5 phage because the KASIA A1-like gene, a counterpart of the pre-early and first injected into the host A1 gene of T5, is located in the replication module, not in terminally repeated sequences. The KASIA phage is also a rare siphovirus with DNA direct RNA polymerase, the hallmark of the *Autographiviridae* family.

Global in silico proteome analyses showed the overall diversity of the *Shewanella* phages, indicating the need for further investigation. Two out of four newly described (pro)phages, i.e., KASIA and MuM16–1, presented themselves as significantly different from the viruses deposited in public databases. Moreover, the KASIA phage could be considered the first of a novel genus with the proposed name *Goldenslopevirus*, within the *Siphoviridae* family. In addition, MuM16–1 expanded the diversity of the known Mu-like phages and gave potential molecular markers for the identification of Mu-like phages integrated in bacterial genomes, and particularly those that could undergo lysogenic-to-lytic conversion after exposure to mitomycin C due to the presence of a S24 peptidase domain in their repressor C proteins.

## Data Availability

Not applicable.

## Supplementary Materials

The following are available online at https://www.mdpi.com/article/10.3390/ijms222011070/s1.
