# Supplementary material for: Genome Study of a Novel Virulent Phage vB_SspS_KASIA and Mu-like Prophages of Shewanella sp. M16 Provides Insights into the Genetic Diversity of the Shewanella Virome
Source: Int J Mol Sci. 2021 Oct 14;22(20):11070. doi: 10.3390/ijms222011070 (PMC8541194; doi:10.3390/ijms222011070)
Supplement: Supplementary file 1 [file ijms-22-11070-s001.zip › Supplementary Figures.pdf]

# Supplementary Materials for

## Genome Study of A Novel Virulent Phage vB_SspS_KASIA and Mu-Like Prophages of *Shewanella* sp. M16 Provides Insights into the Genetic Diversity of the *Shewanella* Virome

**Katarzyna Bujak, Przemyslaw Decewicz, Joanna M. Rosinska and Monika Radlinska***

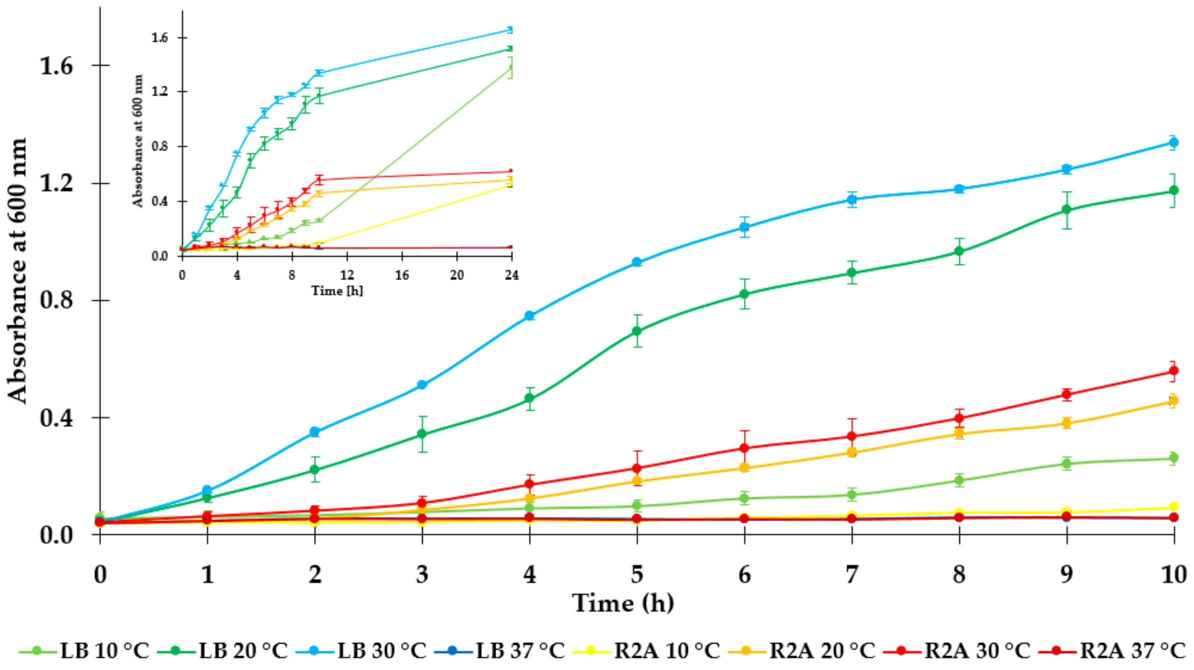


**Figure S1.** Effect of temperature on M16 growth rates, in LB and R2A media, illustrated as optical density curves. The test was carried out in the temperature range 10-37 °C. The *Shewanella* sp. M16 strain was unable to growth at 37°C, but showed the ability to grow at 10, 20 and 30°C. The smaller picture shows the optical density of the M16 cultures after 24 hours.

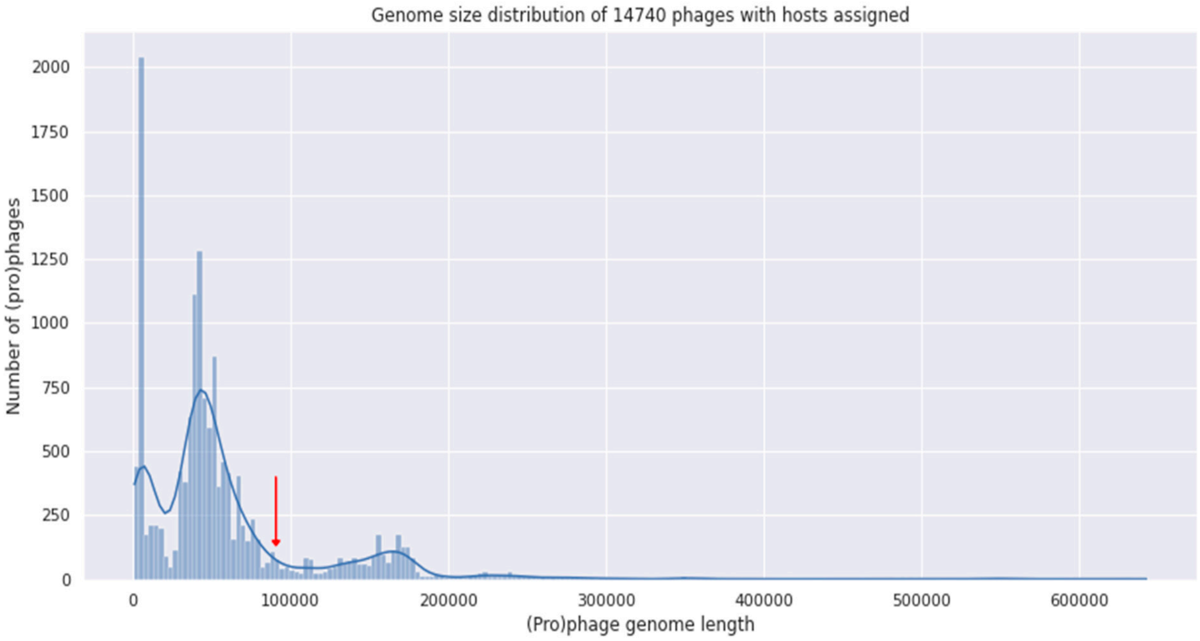


**Figure S2.** Distribution of (pro)phage genome lengths used within this study for comparative analyses. Red arrow indicates the size of the KASIA phage genome.

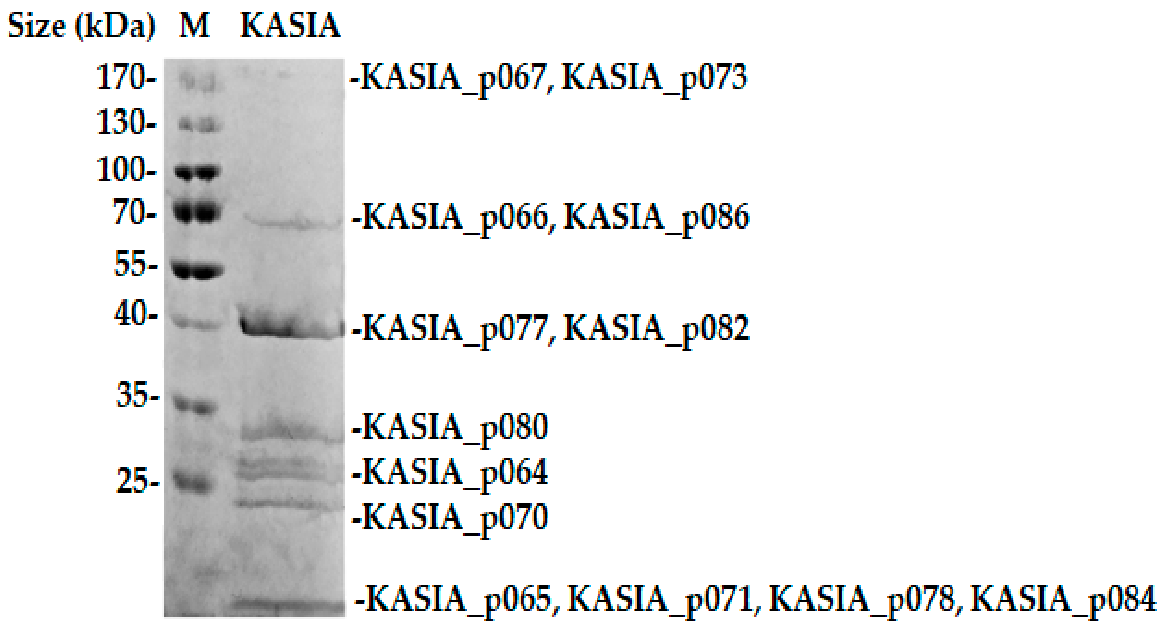


**Figure S3**. KASIA virion proteins separated on a 12% SDS-PAGE gel. Lane M - PageRuler protein ladder SM0671 (Thermo Scientific). Proteins identified by mass spectrometry are shown on the right.

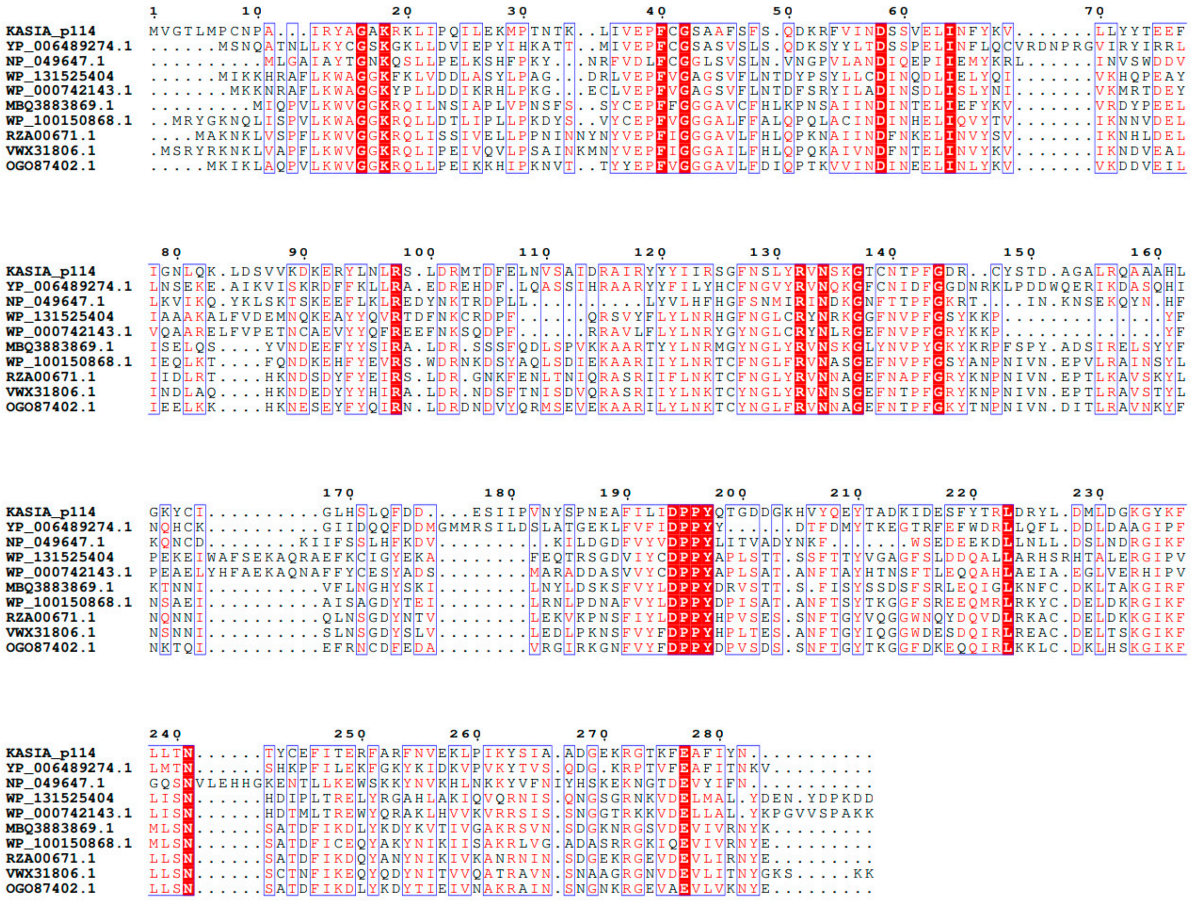

**Figure S4.** Alignment of the experimentally confirmed and putative phage and bacterial Dam-like methyltransferases (MTases) showing similarity to DNA MTase encoded by the KASIA phage (KASIA_p114). The MTases with the following NCBI accession numbers were used for the alignment: YP_006489274.1 (of *Colwellia* phage 9A), NP_049647.1 (of *Escherichia* virus T4), WP_131525404 (of *Shewanella* sp. M16), WP_000742143.1 (of *Escherichia coli* K-12 substr. MG1655), MBQ3883869.1 (of *Succinivibrio* sp.), WP_100150868.1 (of *Snodgrassella alvi*), RZA00671.1 (of *Sphingobacteriaceae* bacterium), VWX31806.1 (of *Moraxellaceae* bacterium 17A) and OGO87402.1 (of *Clostridiales* bacterium GWE2_32_10). The conserved amino acids were distinguished in the following manner: strict identity is visualised via white bold residue with red background, red font represents similarity in groups of residues, i.e. those with similar physico-chemical properties comprising at least 60% of all residues within a column, while blue frames surround columns satisfying at least one of the above conditions.

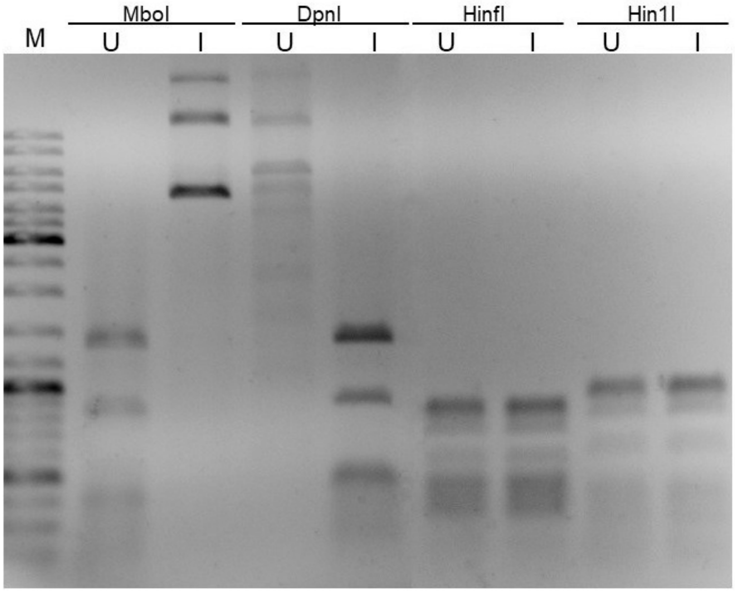


**Figure S5.** Comparative restriction patterns of the pET_KASIAp114 plasmid DNA prepared from *Escherichia coli* ER2929 cells grown in the presence (I) or absence (U) of inducer IPTG and cleaved with selected restriction endonucleases (MboI, DpnI, HinfI or Hin1II). Digest mixtures were electrophoresed on 0.9% agarose gel and stained with ethidium bromide. M—GeneRuler 100–10,000 bp size marker (Thermo Fisher Scientific, Waltham, MA, USA). The plasmid DNAs isolated from the IPTG-induced bacterial culture was cleaved with DpnI (requires adenine methylation of GATC sites for cleavage), but is resistant to MboI digestion (cleavage of GATC sites is inhibited by m6A methylation). In contrast, the pET_KASIAp114 DNA isolated from the noninduced *E.coli* culture was susceptible to MboI but resistant to DpnI. Besides, the DNAs of pET_KASIAp114 isolated from the induced and uninduced cultures were fully digested with the adenine methylation-sensitive endonucleases HinfI and Hin1II used as controls.

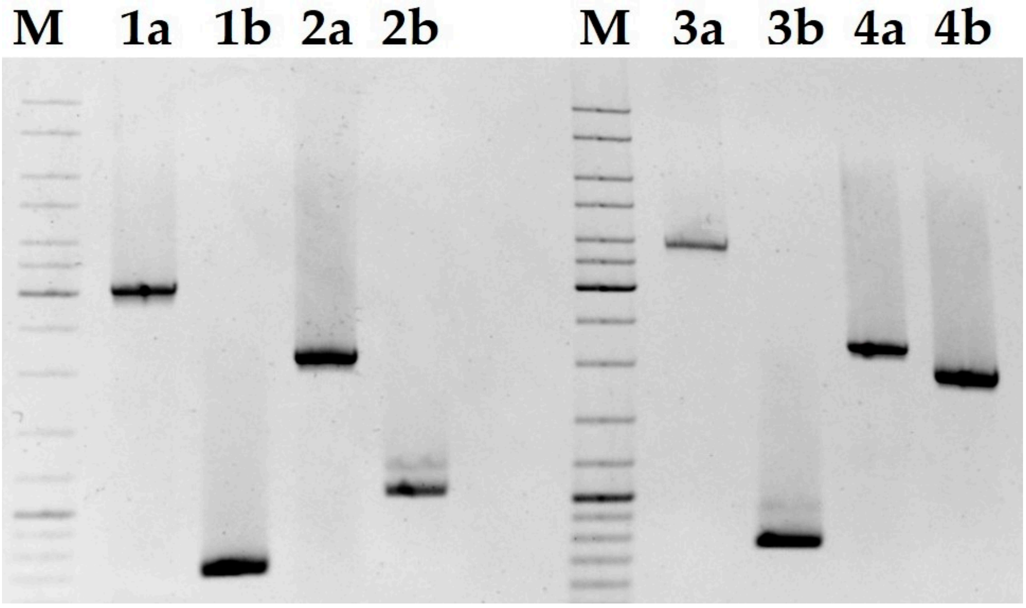


**Figure S6.** Demonstration of the in vivo splicing of the intron DNA interrupting *KASIA_p088* and *KASIA_p100*. Lanes 1a-4a of the 0.8% agarose gel contain PCR products obtained on KASIA DNA with primers Ter1-Ter2, Ter3-Ter5, Pol1-Pol2 and Pol3-Pol5, respectively. Lanes 1b-4b contain PCR products obtained from a cDNA template, using the same primer combinations. Lane M – GeneRuler DNA Ladder Mix 100-10,000 bp size marker (Thermo Fisher Scientific).

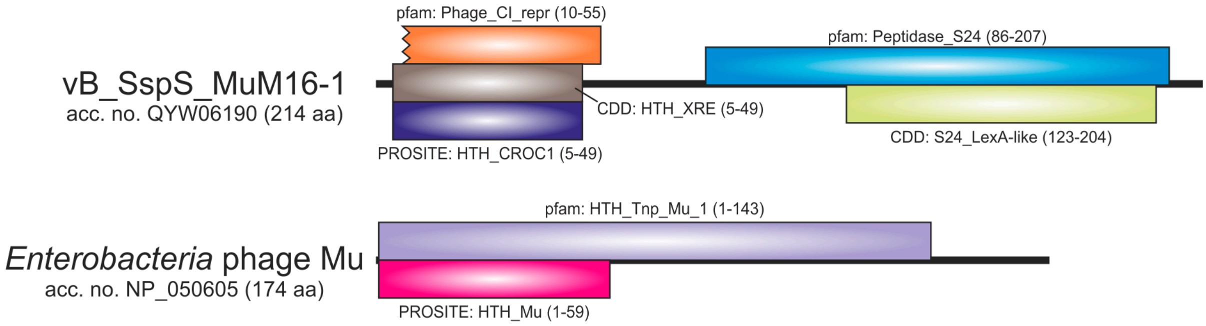


**Figure S7.** Schematic representation of the domains present in the repressor proteins of vB_SspS_MuM16–1 and *Enterobacteria* phage Mu. The lines correspond to the sequence length of both proteins and rectangles indicate the location of domains according to labels on the figure. Domains were identified using InterProScan v 5.52-86.0 [1,2].

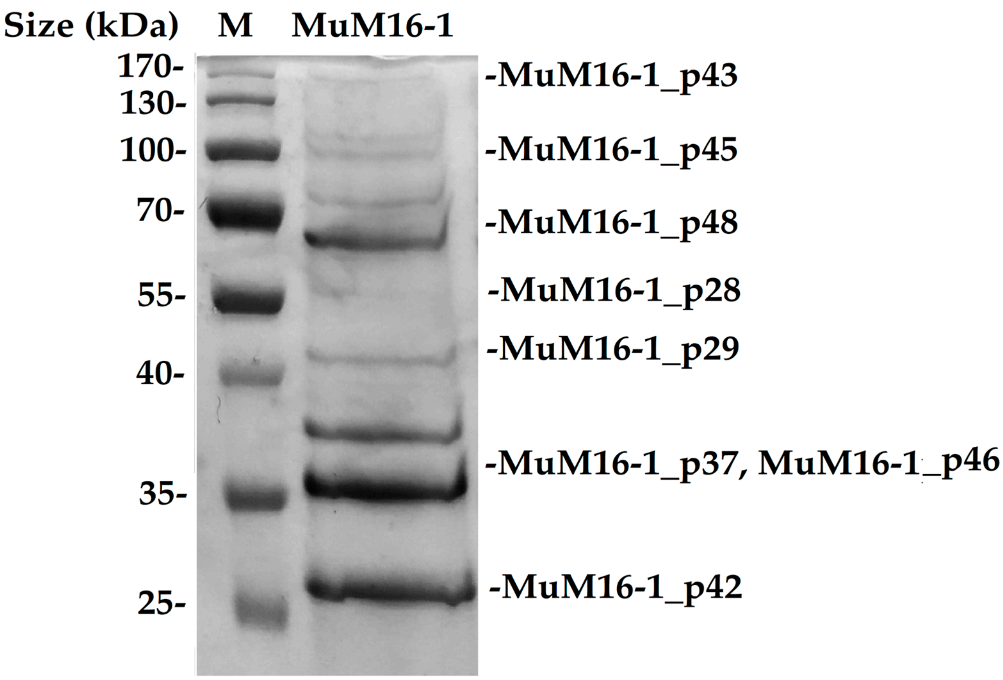


**Figure S8.** The MuM16-1 virion proteins separated on a 12% SDS-PAGE gel. Lane M - PageRuler protein ladder SM0671 (Thermo Scientific). Proteins identified by mass spectrometry are shown on the right.

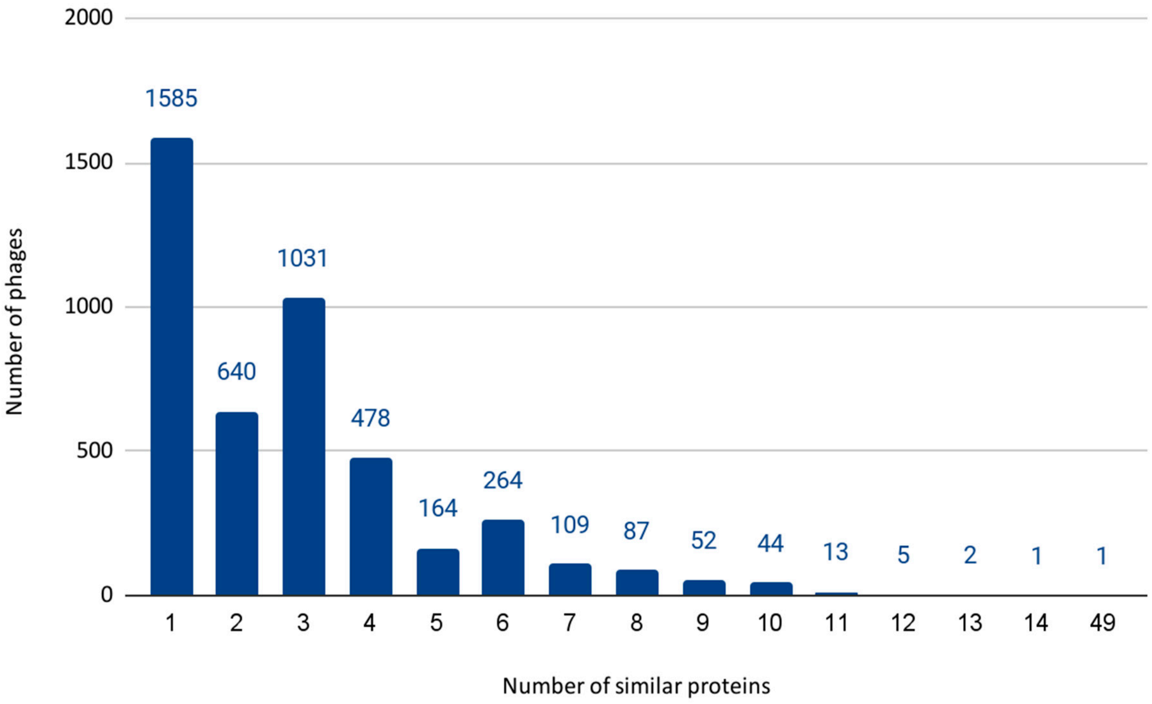


**Figure S9.** Number of phages encoding at least one protein that shows similarity to proteins encoded by the KASIA phage.

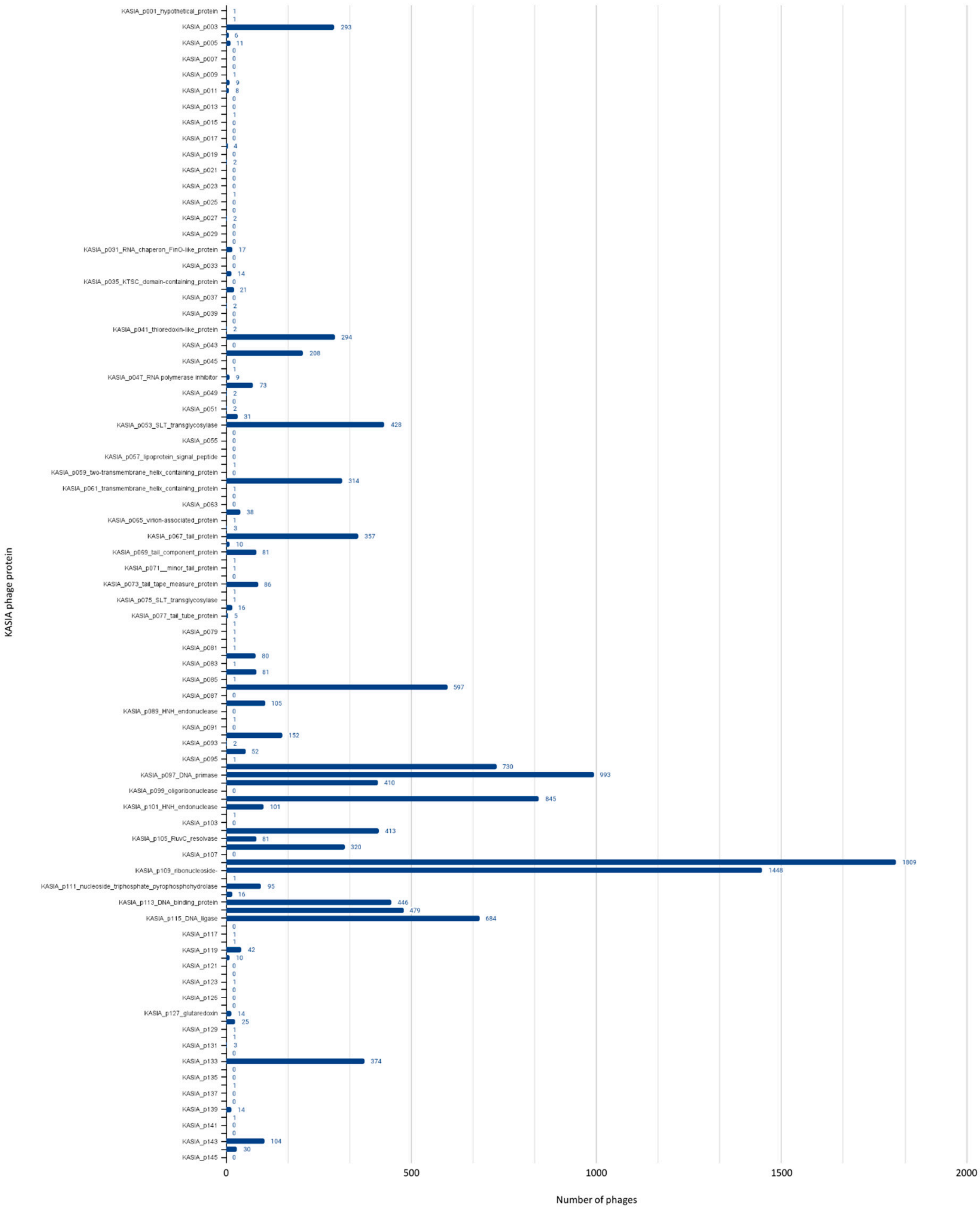


**Figure S10.** Number of proteins encoded by other known bacteriophages of which the complete genomes were deposited in the NCBI database, homologous to the KASIA proteins.

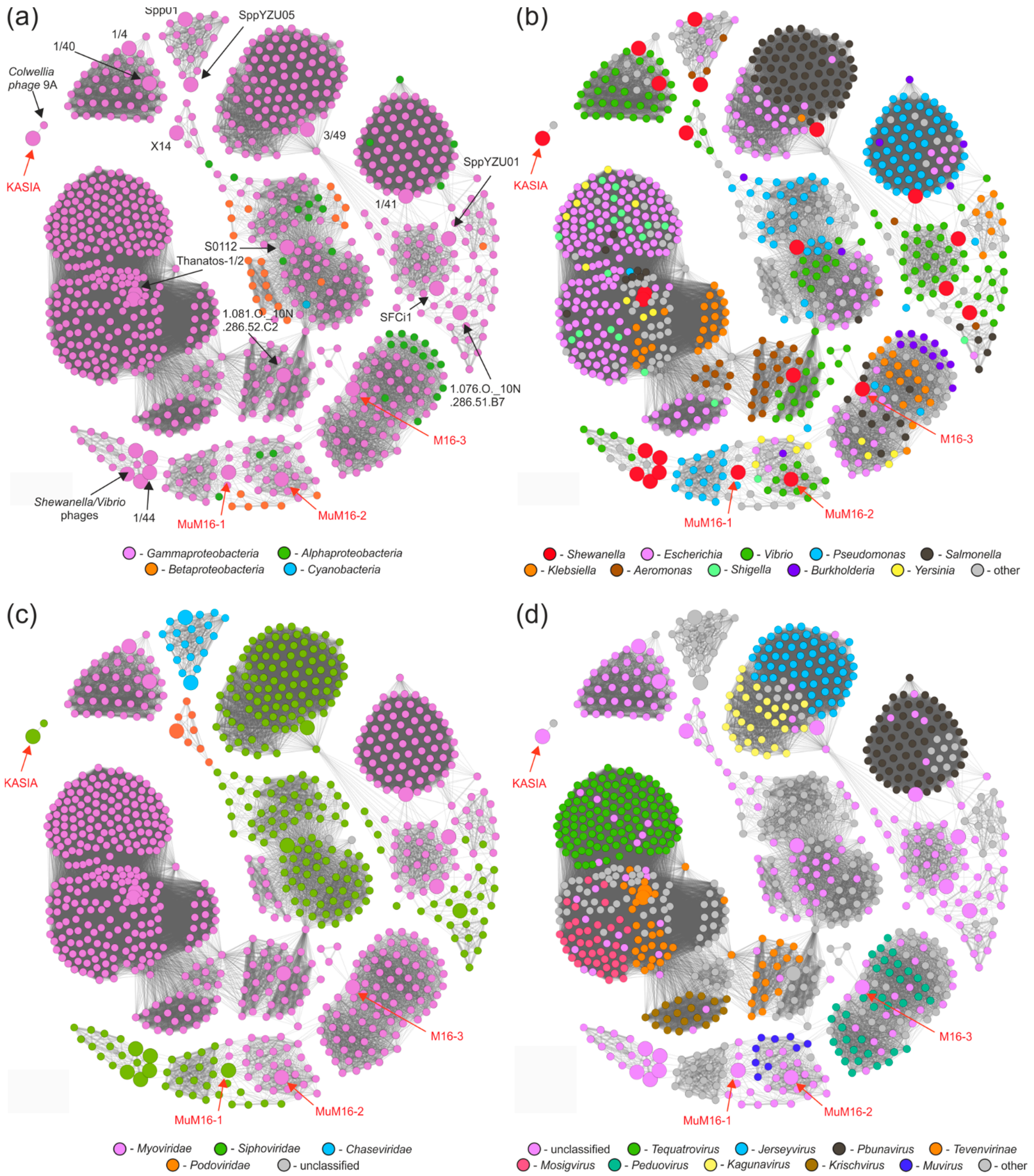


**Figure S11.** Protein-based phage similarity network. Only *Shewanella*--infecting phages whose complete genomes were deposited in the NCBI database and those reflecting significant similarity to these (reflected by the presence of an edge between them) are shown. Nodes representing *Shewanella* phages were enlarged and indicated with arrows. Edge thickness reflects the degree of similarity (the higher the thicker) of proteins encoded by nodes corresponding to a pair of phages. Panels (a) to (d) represent different colouring schemes: (a) - based on phage host class, (b) - based on phage host genus, (c) - based on phage taxonomic classification on family level, (d) - phage taxonomic classification on genus (or in case of *Tevenvirinae*) subfamily level.

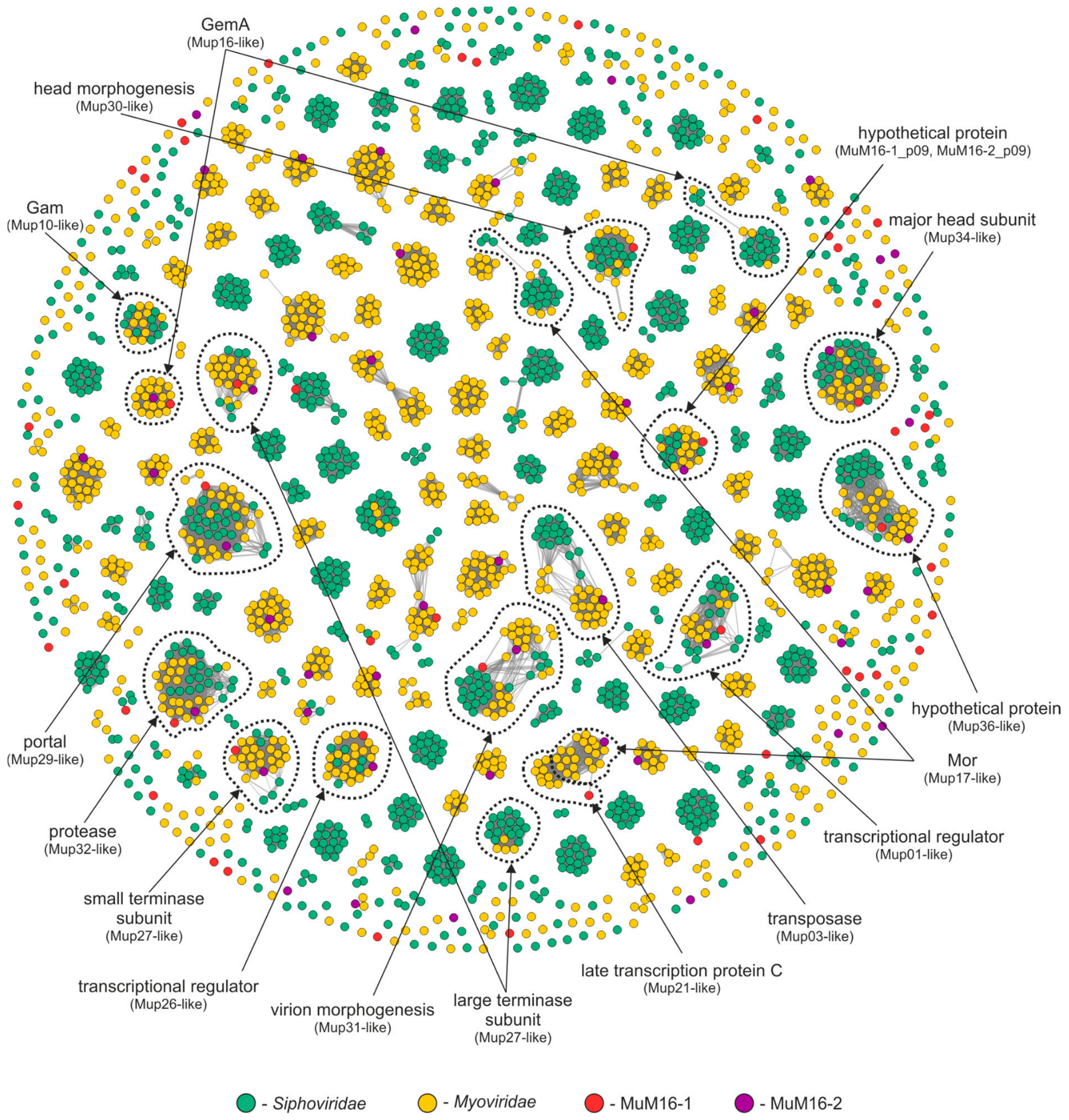


**Figure S12.** Similarity network of predicted proteins encoded by 51 Mu-like phages. The clustering was based on blastp all against all protein search with the following thresholds: e-value of 1e-5 and 85% sequence coverage per HSP. Dashed line was used to indicate clusters of proteins encoded by both sipho- and myoviruses.

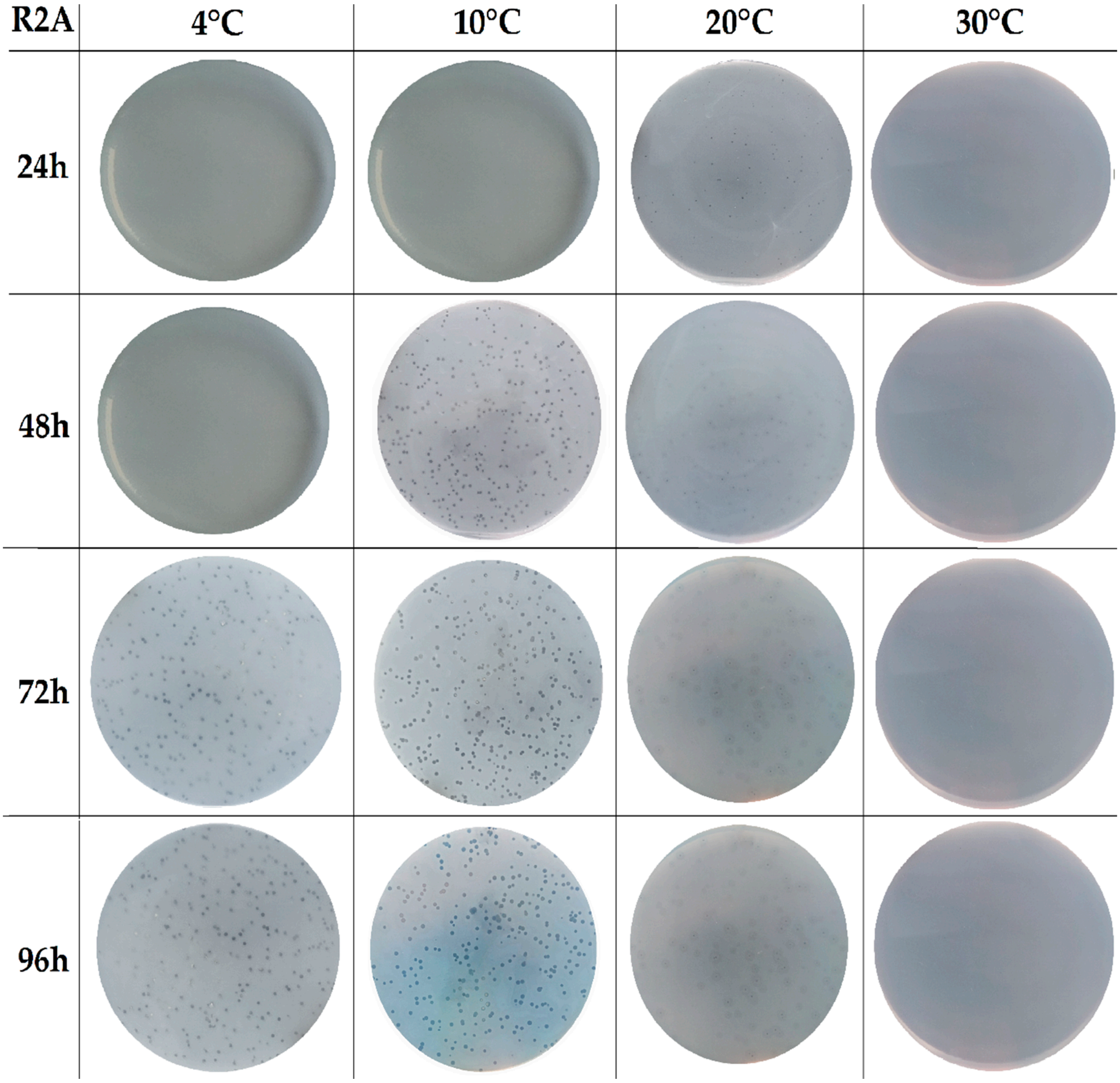


**Figure S13.** The KASIA plaques appearance on M16 cultivated on R2A medium at range of temperatures (4–30 °C) and incubation times. Plates were generated after mixing 100 µL of the KASIA suspension ($10^3$ PFU/ml) with 100 µL of an overnight culture of *Shewanella* sp. M16.

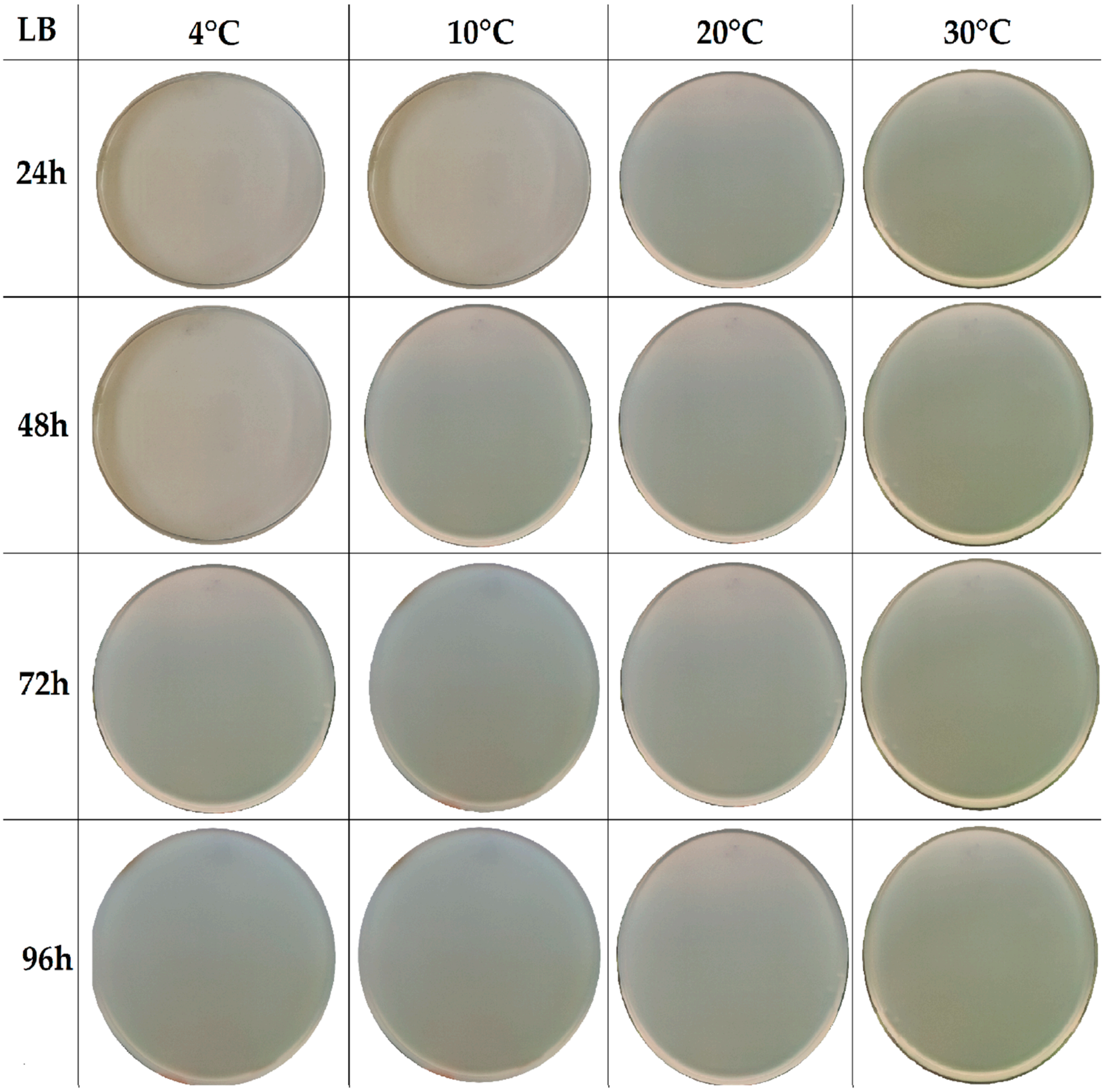


**Figure S14.** Results of the KASIA phage plating on M16 cultivated on LB medium at a range of temperatures (4–30 °C) and incubation times. Plates were generated after mixing 100 μL of the KASIA suspension ($10^3$ PFU/ml) with 100 μL of an overnight culture of *Shewanella* sp. M16.

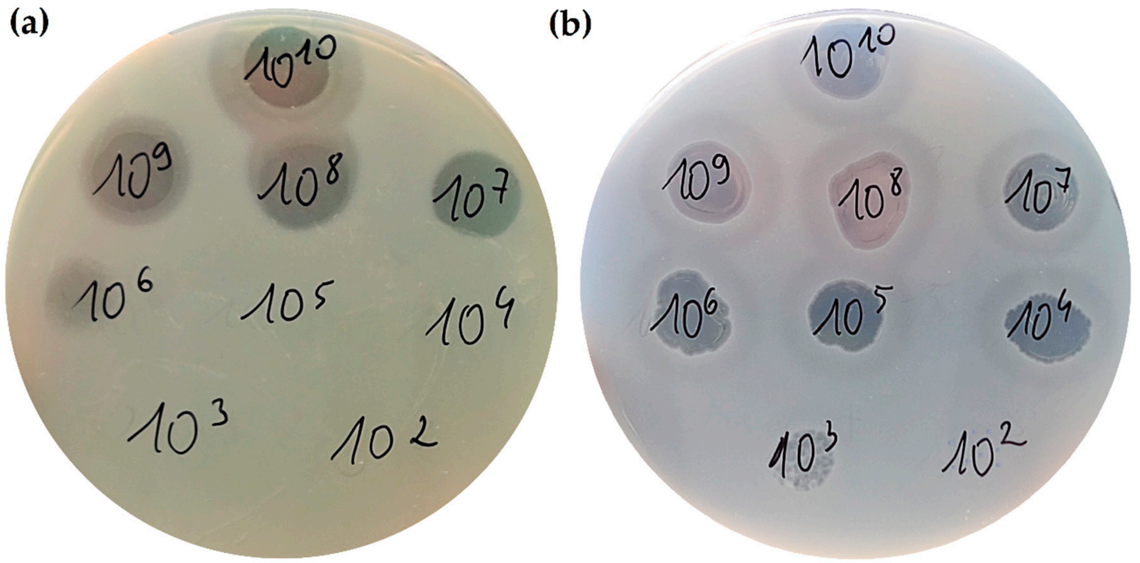


**Figure S15.** Lysis zones of the KASIA phage, generated after the application of 10 µL drops of the KASIA phage suspension with a concentration range of $10^{10}$ to $10^2$ PFU/mL. Plates were incubated for 72 h at 10 °C in **(a)** LB and **(b)** R2A medium.


1. Blum, M.; Chang, H.Y.; Chuguransky, S.; Grego, T.; Kandasaamy, S.; Mitchell, A.; Nuka, G.; Paysan-Lafosse, T.; Qureshi, M.; Raj, S.; et al. The InterPro protein families and domains database: 20 years on. *Nucleic Acids Res* **2021**, *49*, D344-D354, doi:10.1093/nar/gkaa977.
2. Jones, P.; Binns, D.; Chang, H.Y.; Fraser, M.; Li, W.; McAnulla, C.; McWilliam, H.; Maslen, J.; Mitchell, A.; Nuka, G.; et al. InterProScan 5: genome-scale protein function classification. *Bioinformatics* **2014**, *30*, 1236-1240, doi:10.1093/bioinformatics/btu031.
